# Supplementary material for: The diversity and habitat association of medium and large mammals in the Dhidhessa Wildlife Sanctuary, Southwestern Ethiopia
Source: PLoS One. 2025 Mar 21;20(3):e0317441. doi: 10.1371/journal.pone.0317441 (PMC11927904; doi:10.1371/journal.pone.0317441)
Supplement: S1 Appendix — (DOCX) [file pone.0317441.s001.docx]

|  | Riparian forest | Woodland | Savanna grassland | Seasonal flooded grassland |
| --- | --- | --- | --- | --- |
| Taxa_S | 20 | 19 | 19 | 13 |
| Individuals | 732 | 484 | 615 | 188 |
| Dominance_D | 0.1482 | 0.1339 | 0.1239 | 0.147 |
| Simpson_1-D | 0.8518 | 0.8661 | 0.8761 | 0.853 |
| Shannon_H | 2.28 | 2.346 | 2.44 | 2.186 |
| Evenness_e^H/S | 0.4889 | 0.5496 | 0.6036 | 0.6849 |
| Brillouin | 2.222 | 2.267 | 2.372 | 2.062 |
| Menhinick | 0.7392 | 0.8636 | 0.7662 | 0.9481 |
| Margalef | 2.881 | 2.912 | 2.803 | 2.292 |
| Equitability_J | 0.7611 | 0.7967 | 0.8286 | 0.8524 |
| Fisher_alpha | 3.797 | 3.943 | 3.714 | 3.172 |
| Berger-Parker | 0.2609 | 0.2479 | 0.2341 | 0.2394 |
| Chao-1 | 20 | 19 | 19 | 13 |

**S1: Appendix: Supplementary**

**S1 Table: Measuring of biodiversity (Index of diversity in four habitat) in DWS**

**S2 Table: Index of diversity mammals during dry and wet season in DWS**

|  | DRY | Lower | Upper | WET | Lower | Upper |
| --- | --- | --- | --- | --- | --- | --- |
| Taxa_S | 22 | 22 | 22 | 22 | 22 | 22 |
| Individuals | 1093 | 1093 | 1093 | 926 | 926 | 926 |
| Dominance_D | 0.135 | 0.1245 | 0.1456 | 0.1249 | 0.1133 | 0.1366 |
| Simpson_1-D | 0.865 | 0.8544 | 0.8755 | 0.8751 | 0.8634 | 0.8867 |
| Shannon_H | 2.413 | 2.349 | 2.478 | 2.482 | 2.416 | 2.549 |
| Evenness_e^H/S | 0.5077 | 0.4752 | 0.5402 | 0.544 | 0.5082 | 0.5798 |
| Brillouin | 2.366 | 2.302 | 2.43 | 2.428 | 2.362 | 2.493 |
| Menhinick | 0.6654 | 0.6654 | 0.6654 | 0.723 | 0.723 | 0.723 |
| Margalef | 3.001 | 3.001 | 3.001 | 3.074 | 3.074 | 3.074 |
| Equitability_J | 0.7807 | 0.7598 | 0.8016 | 0.8031 | 0.7815 | 0.8246 |
| Fisher_alpha | 3.901 | 3.901 | 3.901 | 4.046 | 4.046 | 4.046 |
| Berger-Parker | 0.2388 | 0.2155 | 0.2621 | 0.2581 | 0.23 | 0.2862 |
| Chao-1 | 22 | 22 | 22 | 22 | 22 | 22 |


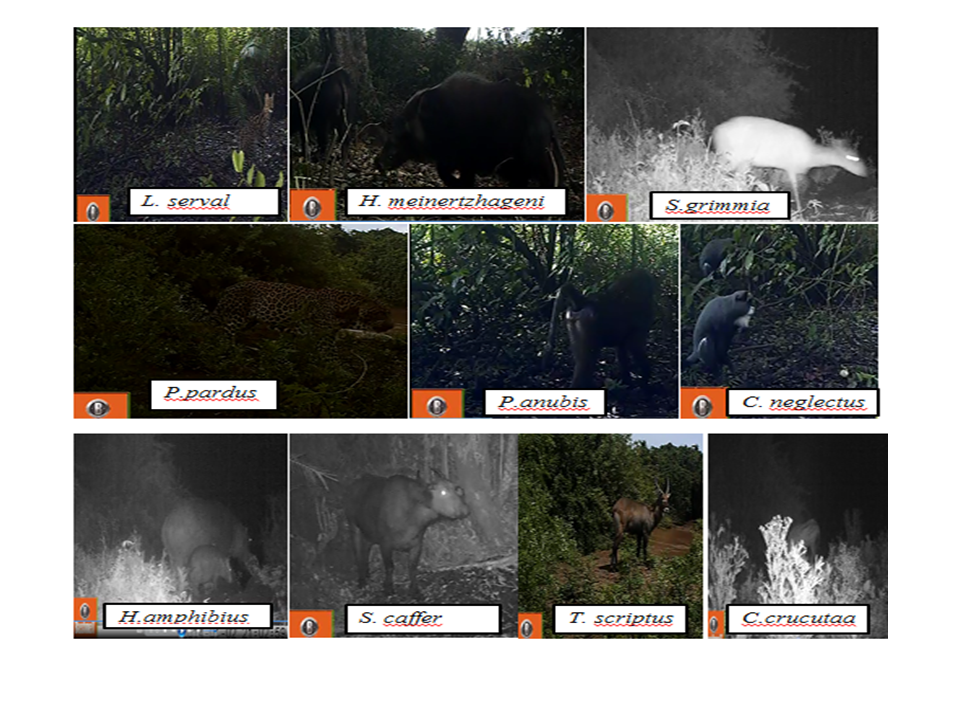
 **S1 Fig: Mammalian species captured by camera trap in DWS**


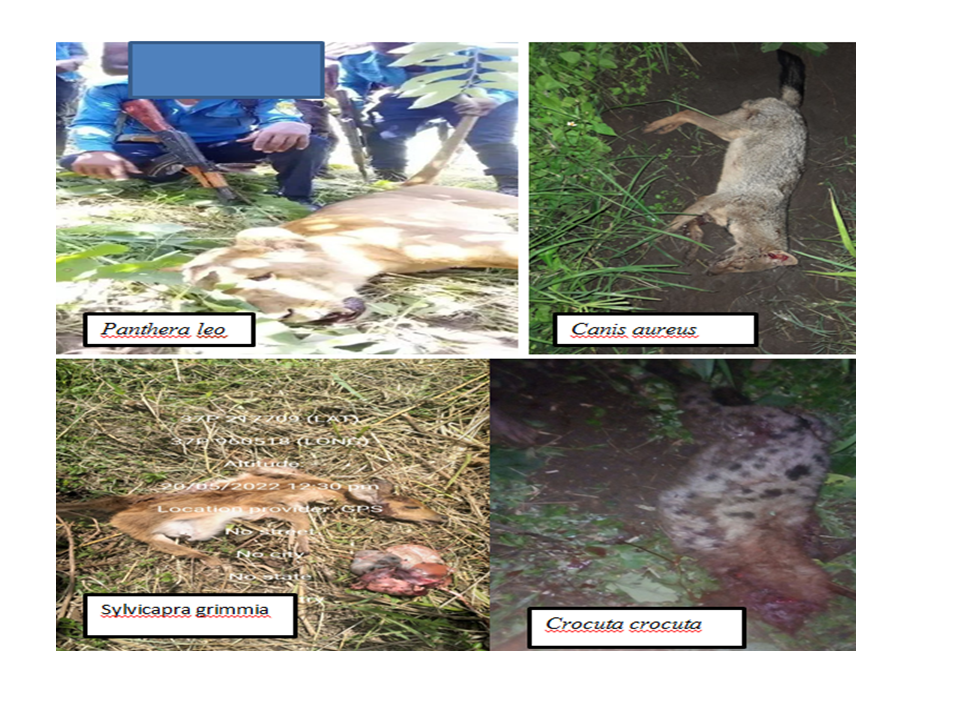
**S 2 Fig: Dead some mammalian species by human activities in the study area**
