# Supplementary material for: The diversity and habitat association of medium and large mammals in the Dhidhessa Wildlife Sanctuary, Southwestern Ethiopia
Source: PLoS One. 2025 Mar 21;20(3):e0317441. doi: 10.1371/journal.pone.0317441 (PMC11927904; doi:10.1371/journal.pone.0317441)
Supplement: Attachment — Submitted filename: PONE-D-24-30486.pdf [file pone.0317441.s003.pdf]

PLOS ONE

The diversity and habitat association of medium and large mammals in the Dhidhessa Wildlife Sanctuary, Southwestern Ethiopia

--Manuscript Draft--

Manuscript Number:	PONE-D-24-30486
Article Type:	Research Article
Full Title:	The diversity and habitat association of medium and large mammals in the Dhidhessa Wildlife Sanctuary, Southwestern Ethiopia
Short Title:	Diversity and habitat association of medium and large mammals in the Dhidhessa Wildlife Sanctuary
Corresponding Author:	Girma Gizachew Tefera, MSC<br>Jimma University College of Agriculture and Veterinary Medicine<br>Jimma, ETHIOPIA
Keywords:	Anthropogenic activities, Dhidhessa Wildlife Sanctuaryy, Diversity , Habitat association.
Abstract:	Understanding species diversity and habitat association is the baseline for developing conservation plan. The study aimed to assess diversity, abundance and habitat association of medium and large sized mammals in the Dhidhessa Wildlife Sanctuary, Southwestern Ethiopia. The survey was conducted from December 2022 to June 2023, considering the wet and dry seasons. Stratified random sampling design was applied to stratify the study area in to four (wooded grassland, riparian forest, seasonally flooded grassland, and savanna grassland) strata based on vegetation and habitat type. Line transects survey, sensor camera trapping, indirect and direct evidence methods were used to collect data during wet and dry seasons. Data were analyzed using chi square test and species diversity indexes. Twenty seven mammalian species were recorded for the area. Order Artiodactyla had the highest number of species (eleven) and with the recorded nine species, Order Carnivora follows. While, orders Rodentia and Tubulidentata each represented by one species. Papio Anubis (n=500, 24.9%) was the most abundant species that is followed by Hippopotamus amphibious (n=364, 17.8%) in the present study area in both wet and dry seasons. But Panthera pardus (n=13, 0.64%) and Civettictis civetta (n=13, 0.64%) were the least abundant species in the present study area. Riverine forest had the highest number of species (n=732, 36.3%) followed by Savanna grassland (n=615, 30.5%). The study revealed that high diversity of species in the area including the rare and threatened species. Therefore, DWS's primary priority should be creating management plans to reestablish the sanctuary as a fully functional sustainable ecosystem and ensuring the social and economic viability of the surrounding community.
Order of Authors:	Girma Gizachew Tefera, PhD Candidate
	Tadesse Habtamu Tessema, Dr
	Tibebu Alemu Bekere, Dr
	Tariku Mekonnen Gutema, Dr
Additional Information:	
Question	Response
Financial Disclosure<br><br>Enter a financial disclosure statement that describes the sources of funding for the work included in this submission. Review the submission guidelines for detailed requirements. View published research articles from *PLOS ONE* for specific examples.	The author(s) received no specific funding for this work.

Powered by Editorial Manager® and ProduXion Manager® from Aries Systems Corporation

This statement is required for submission and **will appear in the published article** if the submission is accepted. Please make sure it is accurate.<br><br>**Funded studies**<br>Enter a statement with the following details:<br>• Initials of the authors who received each award<br>• Grant numbers awarded to each author<br>• The full name of each funder<br>• URL of each funder website<br>• Did the sponsors or funders play any role in the study design, data collection and analysis, decision to publish, or preparation of the manuscript?<br><br>Did you receive funding for this work?	
**Competing Interests**<br><br>Use the instructions below to enter a competing interest statement for this submission. On behalf of all authors, disclose any competing interests that could be perceived to bias this work—acknowledging all financial support and any other relevant financial or non-financial competing interests.<br><br>This statement is required for submission and **will appear in the published article** if the submission is accepted. Please make sure it is accurate and that any funding sources listed in your Funding Information later in the submission form are also declared in your Financial Disclosure statement.<br><br>View published research articles from *PLOS ONE* for specific examples.	The authors declare no conflict of interest

**NO authors have competing interests**<br><br>Enter: *The authors have declared that no competing interests exist.*<br><br>**Authors with competing interests**<br><br>Enter competing interest details beginning with this statement:<br><br>*I have read the journal's policy and the authors of this manuscript have the following competing interests: [insert competing interests here]*<br><br>* typeset	
**Ethics Statement**<br><br>Enter an ethics statement for this submission. This statement is required if the study involved:<br><br>• Human participants<br>• Human specimens or tissue<br>• Vertebrate animals or cephalopods<br>• Vertebrate embryos or tissues<br>• Field research<br><br>Write "N/A" if the submission does not require an ethics statement.<br><br>General guidance is provided below. Consult the submission guidelines for detailed instructions. **Make sure that all information entered here is included in the Methods section of the manuscript.**	Jimma Univesristy

**Format for specific study types**<br><br>**Human Subject Research (involving human participants and/or tissue)**<br>• Give the name of the institutional review board or ethics committee that approved the study<br>• Include the approval number and/or a statement indicating approval of this research<br>• Indicate the form of consent obtained (written/oral) or the reason that consent was not obtained (e.g. the data were analyzed anonymously)<br><br>**Animal Research (involving vertebrate animals, embryos or tissues)**<br>• Provide the name of the Institutional Animal Care and Use Committee (IACUC) or other relevant ethics board that reviewed the study protocol, and indicate whether they approved this research or granted a formal waiver of ethical approval<br>• Include an approval number if one was obtained<br>• If the study involved *non-human primates*, add *additional details* about animal welfare and steps taken to ameliorate suffering<br>• If anesthesia, euthanasia, or any kind of animal sacrifice is part of the study, include briefly which substances and/or methods were applied<br><br>**Field Research**<br><br>Include the following details if this study involves the collection of plant, animal, or other materials from a natural setting:<br>• Field permit number<br>• Name of the institution or relevant body that granted permission	
**Data Availability**<br><br>Authors are required to make all data underlying the findings described fully available, without restriction, and from the time of publication. PLOS allows rare exceptions to address legal and ethical concerns. See the PLOS Data Policy and FAQ for detailed information.	Yes - all data are fully available without restriction

A Data Availability Statement describing where the data can be found is required at submission. Your answers to this question constitute the Data Availability Statement and **will be published in the article**, if accepted.<br><br>**Important:** Stating 'data available on request from the author' is not sufficient. If your data are only available upon request, select 'No' for the first question and explain your exceptional situation in the text box.<br><br>Do the authors confirm that all data underlying the findings described in their manuscript are fully available without restriction?	
**Describe where the data may be found in full sentences. If you are copying our sample text, replace any instances of XXX with the appropriate details.**<br><br>• If the data are **held or will be held in a public repository**, include URLs, accession numbers or DOIs. If this information will only be available after acceptance, indicate this by ticking the box below. For example: *All XXX files are available from the XXX database (accession number(s) XXX, XXX.).*<br>• If the data are all contained **within the manuscript and/or Supporting Information files**, enter the following: *All relevant data are within the manuscript and its Supporting Information files.*<br>• If neither of these applies but you are able to provide **details of access elsewhere**, with or without limitations, please do so. For example:<br><br>*Data cannot be shared publicly because of [XXX]. Data are available from the XXX Institutional Data Access / Ethics Committee (contact via XXX) for researchers who meet the criteria for access to confidential data.*<br><br>*The data underlying the results presented in the study are available from (include the name of the third party*	All relevant data is within the paper and it's supporting information files

and contact information or URL).<br>• This text is appropriate if the data are owned by a third party and authors do not have permission to share the data.<br><br>* typeset	
Additional data availability information:	Tick here if the URLs/accession numbers/DOIs will be available only after acceptance of the manuscript for publication so that we can ensure their inclusion before publication.; Tick here if your circumstances are not covered by the questions above and you need the journal’s help to make your data available.

Manuscript Click here to access/download;Manuscript;Mammals manuscript.docx

The diversity and habitat association of medium and large mammals in the Dhidhessa Wildlife Sanctuary, Southwestern Ethiopia

Girma Gizachew Tefera[1], Tadesse Habtamu Tessema[2] , Tibebu Alemu Bekere[3], Tariku Mekonnen Gutema[4]

[1] Department of Natural Resource Management, Colleague of Agriculture and Veterinary Medicine, Jimma University, Jimma, Ethiopia.

[2] Department of Biology, College of computational Sciences, Jimma University, Jimma, Ethiopia

[3] Department of Natural Resource Management, Colleague of Agriculture and Veterinary Medicine, Jimma University, Jimma, Ethiopia.

[4] Department of Natural Resource Management, Colleague of Agriculture and Veterinary Medicine, Jimma University, Jimma, Ethiopia.

Corresponding author: kennaa20047@gmail.com
Tel: +251917550062

Abstract

Understanding species diversity and habitat association is the baseline for developing conservation plan. The study aimed to assess diversity, abundance and habitat association of medium and large sized mammals in the Dhidhessa Wildlife Sanctuary, Southwestern Ethiopia. The survey was conducted from December 2022 to June 2023, considering the wet and dry seasons. Stratified random sampling design was applied to stratify the study area in to four (wooded grassland, riparian forest, seasonally flooded grassland, and savanna grassland) strata based on vegetation and habitat type. Line transects survey, sensor camera trapping, indirect and direct evidence methods were used to collect data during wet and dry seasons. Data were analyzed using chi square test and species diversity indexes. Twenty seven mammalian species were recorded for the area. Order Artiodactyla had the highest number of species (eleven) and with the recorded nine species, Order Carnivora follows. While, orders Rodentia and Tubulidentata each represented by one species. *Papio Anubis* (n = 500, 24.9%) was the most abundant species that is followed by *Hippopotamus amphibious* (n = 364, 17.8%) in the present study area in both wet and dry seasons. But *Panthera pardus* (n=13, 0.64%) and *Civettictis civetta* (n=13, 0.64%) were the least abundant species in the present study area. Riverine forest had the highest number of species (n=732, 36.3%) followed by Savanna grassland (n=615, 30.5%). The study revealed that high diversity of species in the area including the rare and threatened species. Therefore, DWS's primary priority should be creating management plans to reestablish the sanctuary as a fully functional sustainable ecosystem and ensuring the social and economic viability of the surrounding community.

Key word: Anthropogenic activities, Dhidhessa Wildlife Sanctua, Diversity, habitat association.

Introduction

Background of the study

Mammals are a remarkable category due to their diversity in species, morphologies, ecologies, and behaviors [1]. The class Mammalia contains 5487 species globally. On the African continent, there are more than 1150 distinct mammalian species [2,3] , with 360 of those species found in eastern Africa [2], and 320 in Ethiopia [4-6] . Fifty-five of the 320 mammalian species known to exist in Ethiopia are endemic [6-7]. Mammalian species support the conservation efforts of other species [8-9], and maintain the ecological balance of terrestrial ecosystems through grazing, predation, and seed dispersal. As a result, they function as umbrella species in the context of ecology [10-11].

Mammals offer significant benefits to humans in terms of the economy, including food and revenue [12]. In contrast, anthropogenic activities that are not appropriate have put mammalian species in danger of extinction both locally and worldwide [13-14]. Nowadays, there are several issues facing mammalian species around the world, especially in developing countries like Ethiopia: habitat degradation due to overgrazing, settlement, hunting, and expanding agriculture [15-17]. provide detail evidence on threats and level of impacts from global - local

There exists a high correlation between the types of habitat occupied by different mammalian species within an ecosystem in many forests [18]. In general, larger ecosystems have more diverse microhabitats and varied vegetation structures. These microhabitats and differing resources are available to species with different feeding and microhabitat requirements, as supported by [3]. In varying seasons and habitat types, mammals are not uniformly dispersed [19]. The main cause of this is that each species has a unique range that is the outcome of the

interaction between the ecological conditions of the present day and the species' evolutionary history. However, many species have similar, though not always identical, distribution patterns. Conversely, individual distribution within a region of easily accessible habitats is indicated by home ranges, territories, and microhabitats [18]. Access to essential resources like food, housing, and water availability determines these. Moreover, changes in the ecosystem's climate, competition, predation, hunting patterns, and habitat all affect the limits of a species' range [20]. As to [7], medium- and large-sized mammals have heightened sensitivity to alterations in their habitat, rendering them dependable markers of the environmental condition. Structurally complex ecosystems may provide more niches and diverse methods of utilizing natural resources, increasing species diversity [21].

According to a study, medium- and large-sized mammals make up more than 60% of Ethiopia's mammal species [22]. The weight of a medium-sized mammal ranged from 2 to 7 kg, whereas that of a large mammal was greater than 7 kg [23]. The diversity of wildlife species found in the Dhidhessa Wildlife Sanctuary is not supported by scientific evidence or official documentation, but numerous oral histories and reports from local informants attest to the abundance of wildlife, particularly mega herbivores and large carnivores, and support the sanctuary's designation from fifty years ago. The country's wildlife resources, including those found in well-established protected areas, have been severely depleted over the past thirty years for a variety of reasons. These include civil war, widespread settlement in the then-wilderness areas, such as the Dhidhesa wildlife sanctuary, widespread settlement during periods of drought, and the unstable and quickly changing central government [7, 24]. Because of the extensive anthropogenic activity around the Dhidhessa River, a greater portion of the wildlife habitats have been destroyed far earlier than the wildlife resources in the area have been documented. This poses a

threat to wildlife habitats,. In addition, the Arjo Dhidhessa sugar plant is the result of the conversion of nearby regions surrounding the sanctuary into automated sugarcane farms [24]. Therefore, the current study was conducted at DWS, one of the wildlife sanctuaries in southwest Ethiopia, where scientific data are essential for studying the diversity and habitat association of medium- and large-sized mammals. These studies serve as the foundation for additional conservation and management strategies. What is the objective of this study?

The study area and methods

Description of the Study Area

The study was conducted in Dhidhessa Wildlife Sanctuary (DWS) and its surrounding areas in southwestern Ethiopia. ~~The Sanctuary is located between 8^{0}30' and 8^{0}40' N latitude and between 36^{0}22' and 36^{0}43' E longitude and covers a total area of 1300 km^{2}~~. The sanctuary is about 395 km southwest of Addis Ababa (the capital city) ~~and shared among three administrative zones (East Wollega, Buno Bedele, and Jimma), all within Oromia National Regional State~~ (Fig. 1). The altitude within the sanctuary ranges between 1350 and 1050 masl. The sanctuary has two different seasons, dry (November to February) and wet (June to September), with uni-modal rainfall ranging from 648 mm to 2001.8 mm. The sanctuary experienced relatively warm temperatures, with mean annual minimum and maximum temperatures of 12°C and 35°C [25]. In addition to the vast lowland plains along the Dhidhessa river bank, the land form of the sanctuary includes undulating hilly terrain and mountains surrounding the sanctuary from the north and western sides. Habitats in the study area include wooded grassland, riparian forests, seasonal flooded prairies, and extensive savannah grassland. This region was once home to a variety of wildlife species (especially antelopes, mega herbivores, and large carnivores). For the conservation of the habitats and the large mammals therein, the Ethiopian government

the map tells this

designated the DWS in 1970. Before the management system of the sanctuary was well established, for various reasons (including the political instability that spans over three decades and the extensive settlement program for people displaced from the north and eastern parts of the country), part of the then wildlife areas were taken and other parts were heavily encroached. In addition, the extensive plain area adjacent to the sanctuary from the northeastern side was used for the mechanized sugarcane farm to produce raw materials for the state-owned Arjo Dhidhessa Sugar Factory (ADSF). As a result, some evaluative reports show that the DWS prematurely hindered itself from achieving the objectives for which it was established [24].

Methods

A preliminary survey was carried out in February 2021 in the study area to secure information such as the climatic conditions, habitat types, topographic information, level of anthropogenic activities, etc. The Dhidhessa Wildlife Sanctuary (DWS) was characterized by heterogeneous habitats. Based on the vegetation types, the habitat types of the study area were classified into four (riparian forest, savanna grassland, seasonally flooded grassland and wooded grassland) [2,26]. During the preliminary survey, sampled study sites were determined (Table1). incomplete idea Transect lines counts were used to determine the diversity, and habitat association of medium and large sized mammals. Transect lines was placed on the stratified habitat types in a random fashion and proportional to an area of the habitat type [27]. A total of 25 lines transects, covering about 25% (24.65 Km^2) of the study area. Wildlife trails, footpaths, and other access routes were also used as transect lines. The average length of line transect was 5 km and the width were determined by the vegetation type and season, and ranges between 75m (riverine and tall grass bearing wooded grassland) and 200 m (open grasslands) and each transect ends about a km from the habitat edge [28]. Adjacent transect lines were spaced between one and two Km.

Data collection was conducted once a month for a total of 6 months during both the dry seasons (December, January and February, 2022) and the wet seasons (April, May and June, 2022) to assess the diversity, and habitat association of medium and large sized mammals of DWS. Mammal survey was carried out for three days per season and two times per day 8:00 a.m.to 10:00 a.m. in morning and 4:00 p.m. to 6:00 p.m. late afternoon, when most diurnal medium and large mammals were more active in the study area [29-30]. Each transect line of each habitat type was visited once for a month and at the same period (early morning and evening). During data collection the researcher and 24 assistants person were walking on foot along transect line established and directly count all the individuals of medium and large mammals sighted with their respect species using naked eye and digital camera [31]. Upon detection of animals; habitat types, season of the year, species name and number of individuals observed was record. For identification of mammal species, local expertise and *Kingdon Field Guide to African Mammals* [32] was used.

During the line transect survey, indirect evidences such as Bushnell wildlife camera traps, scats, foot print & hoof and pug marks, hair remains, characteristic burrows, kill remains, etc., were also used to determine the presence/absence of the species and their relative abundance [33]. All basic information of the observed mammals (the species, group size, habitats types, the season, etc.) was recorded.

Data analysis

~~All data collected during the study period were presented in a table by season and habitat type.~~ The Shannon–Wiener Diversity Index was used [34] to calculate the distribution, abundance, and evenness of species between the wet and dry seasons and within habitat types, and SPSS version 20 software was used to analyze the data. Excel version 2016 was also used to generate the

relative abundance and species diversity indexes using formatted formulas. Moreover, the conservation status of mammals identified from DWS was described as vulnerable and least concerned as per the IUCN Red List [35]. A Chi-Square ($\chi 2$) check, at 0.05 degree of significance might be used to evaluate differences in the abundance of mammal species between habitats and the standard seasonal distinction in the abundance of medium and large-sized mammal species in the examination vicinity.

Results

Species composition and richness

In the study area, during the dry and wet seasons, a total of 27 species of medium- and large-sized animals from five orders and twelve families were identified (Table 2). Of these, 1511 photographs taken with a camera trap over the research periods demonstrated the presence of 11 animal species (S1Fig.). Five orders—Primate, Artiodactyla, Carnivora, Rodentia, and Tubulidentata—were used to group the recorded mammals. With eleven species, the order Artiodactyla has the most of all the orders, followed by the order Carnivora with nine species. Conversely, one species each represented the orders Rodentia and Tubulidentata. With seven species, the Bovidae family was the largest family at the family level, followed by the Cercopithecidae family. On the other hand, only one species represented each of the six families. Of the 27 species of medium- and large-sized mammals found in the current study area, five species—the crested porcupine (*Histrix cristata*), honey badger (*Mellivora capensis*), black-blacked jackal (*Canis mesomelas*), common jackal (*Canis aureus*), and aardvark (*Orycteropus afer*)—accounted for 18.5% of the total. The remaining 22 species, or 81.5 %, were large mammals (Table 2).

Species relative abundance and conservation status of mammals

There were significant variations in the seasonal species abundance of medium- and large-sized mammals, with a total of 2019 individuals reported, of which 1093 (54.1%) were observed during the dry season and 926 (45.9%) individuals during the rainy season ($\chi2 = 13.81$, df = 1, $p<0.05$). *Papio anubis* represented (n = 500, 24.9%) was the most abundant species individuals recorded followed by *Hippopotamus amphibious* (n = 364, 17.8%). On the other hand, *Civettictis civetta* (n = 13, 0.64%) and *Panthera pardus* (n = 13, 0.64%) were the two least abundant species (Table 3).

Out of the 27 species of mammals identified in DWS, 11 (40.7%) were considered occasional, 5 (18.5%) frequent, 4 (14.8%) common, and 7 (25.9%) rare. Comparably, Table 4 demonstrates that of the known mammalian species, about 70.4% were categorized as least concern and 14.8% as vulnerable. However, the remaining 14.8% are thought to be conservation-dependent, near threatened, and at lower risk (Table 4).

Habitat association of mammals

In the research area, seasonal variations in the distribution of medium and large mammal species were noted (Table 5). For example, mammals regularly used riparian forests and savanna grasslands in both wet and dry seasons. In contrast, animals used the seasonal flooded grassland (9.3%) less frequently in both seasons (Table 5). There was a noticeable difference in the distribution of indicators linked to mammals' observations in various habitat types during the wet and dry seasons ($\chi2 = 18.87$, df = 3, $p < .05$).

Species diversity and similarity indices

Table 6 shows that compared to other habitats, the Shannon diversity of mammal species was higher in the savanna grassland (H = 2.44) and forest (H = 2.35). Conversely, seasonal flooded

grasslands had the lowest species diversity (H′=2.18).Nonetheless, there was no significant variation in the Shannon-Wiener Index values among the four habitat types. Seasonally flooded grasslands (E = 0.6849) and riparian forests (E = 0.4889) showed the highest and lowest evenness of the mammalian species, respectively. On the other hand, in the riparian forest (D = 0.1482) and the savanna grassland (D = 0.1239), the highest and lowest dominance of mammalian species were observed (S1 table). Additionally, during the dry and wet seasons, the dominance of mammalian species and the Shannon different characteristics record were comparable (S2 table). The research area's maximum species diversity (0.8761) was shown by Simpson's index of diversity.

Discussion

Diversity and relative abundance

The topography, climate, and vegetation types of the current research area have produced a variety of habitats for medium- and large-sized animal species. For example, the 27 species of medium- and large-sized mammals found in the current study area had a significantly higher diversity than the 23 species of mammals found in Arawale National Reserve, Kenya,[36], the 23 species found in the Baroye control hunting area [37], the 22 species found in the fragmented remnant forest near Asella town [18], , a total of 18 species were observed by [2], around the Wondo Genet forest area, 14 species of medium- and large-sized mammals were recorded in Yayu forest by [38], and 12 species of mammals were identified in the Wabe forest's fragments by [29]. A higher level of mammalian diversity in the current research area could have been caused by sufficient vegetation cover and resource availability. Direct observations were also used to identify the majority of the animal species found in the current research area. [23]

revealed that the Watch protected forest produced the same results. The fact that the habitats are so open, maybe as a result of habitat loss and fragmentation, allows for the detection of mammals with the unaided eye. The current study region has a high degree of secretiveness, illusiveness, shyness, and human disturbance, which contributes to the low number of large mammal species that were identified by indirect evidence rather than direct sighting. This outcome was comparable to research conducted by [2] in Loka Abaya National Park and by [29] from Wabe forest fragments.

In contrast to the high diversity of animals (41) documented in Gambella National Park, Ethiopia [39] and 31 species recorded from Dhati Wolel National Park, Ethiopia [40] the results showed comparatively lesser diversity in the study area. The lower diversity of mammals observed in the current study area could be attributed to fragmentation caused by many anthropogenic activities, severe habitat loss, conflict between humans and wildlife, or low level of attitude among local residents toward conservations mammals [24]. Similarly, [41] noted that the demand for wide areas, overexploitation, and habitat fragmentation have led to decline in medium- and large-sized mammals.

The orders Artiodactyla (11 species) and Carnivora (9 species) were the most abundantly seen mammal groups in the current study region. The findings of [40] in Dhati Wolel National Park are consistent with the high number of Artiodactyla in the current research region. With the largest diversity of ungulates on Earth, the African savanna environment has supported multispecies animal production systems for thousands of years [42]. The large proportion of riverine habitats and periodically flooded grasslands that supplied enough grass for grazing during the dry season was probably responsible for the high diversity of herbivores in the current study area. In addition, during the dry season, fires occur in the savanna grassland that is dotted

with Combertum-Terminalia woods. This would enable the high-nutrient new grasses to regenerate, supporting a wide variety of grazers throughout the wet season. Rainfall, fire and herbivory are the prime driving variables in African savanna ecosystems [42]. On the other hand, a large number of different and prolific herbivore species are thought to be responsible for the high diversity of carnivores seen in DWS. Large groups of herbivores, including hippopotamus and African buffalo, were regularly seen throughout the current study, suggesting their high abundance in the region and their ability to serve as a plentiful source of prey for a variety of large carnivore species [43].

Relative abundance

In the current study area, during both the wet and dry seasons, *Papio anubis* was the species with comparatively higher abundance. Nevertheless, the two least common species were *Civettictis civetta* and *Panthera pardus*. Given that the species is suited to eating a wide range of foods and endure various climatic and topographic differences, this may have something to do with its feeding habits. Similar finding was reported by [41] in Birbir protected forest western Ethiopia. In contrast, carnivores were the least common in the study area in terms of both number and distribution. According to respondents, the presence of illegal hunting as a result of widespread livestock predation by carnivores (especially spotted hyena and common jackal) in and around the research area significantly lowers their population. Because of human interference with their cryptic and nocturnal behaviors, it was difficult to document their presence. Similarly, least abundance of carnivores also reported in Dati Wolel National park [7].

~~Additionally,~~ despite the fact that the area is surrounded by a landscape that is dominated by humans and frequently poses a threat to their survival, many mammals have the ability to transition from diurnal to nocturnal or crepuscular activity. In the riverine forest, however,

nonhuman monkey species were frequently sighted. This could be because nonhuman primates have successfully adapted to human disturbance and changing environments. Similar finding was reported by [44] in the Jorgo-Wato Protected Forest in Western Ethiopia.

Habitat association of mammals

Mammals in the current research region select one habitat over another depending on a variety of factors, including soil types, topography, water availability, and the composition, quality, and availability of feed [16, 23, and 45]. In the research area, riverine forest supported a large number of mammal species during both seasons, followed by savanna grassland. Seasonally flooded grasslands sustained the least amount of diversity. The abundance of food, water, and vegetation cover may be the cause of this high observation rate, but the lack of refuges and vegetation cover may be the reason for the low number of mammal individuals. Similar result was reported by [16, 23, and 41]

Species diversity indices

The richness of species in the various ecosystems varies, as indicated by the species diversity index. The savanna grassland in the current study region, for instance, has the highest species diversity (H′=2.44), while the seasonal flooded grassland has the lowest species diversity (H′=2.18). Likewise, studies by [18] showed that variables like habitat quality, species preferences, availability of food resources, dense forest cover, and water are related to variations in the distribution and abundance of mammal species within different habitats, and that these variables are likely to lead to higher species richness. Our findings go counter to those of the study by [23.46], which indicated that the natural forest had a greater species variety. Human pressure on DWS is one potential source of these variations [24].

Conclusion and recommendations

The current study provided baseline data regarding the presence and abundance of medium- and large-sized animal species in the DWS, southwestern Ethiopia, as well as information about numerous anthropogenic activities. DWS is unique in that it is home to animals with great conservation value, including large mammals like *Sincerus caffer* and *Hippopotamus amphibibius*, and globally threatened species of mammals like *Panthera leo* and *Panthra pardus*, which together indicate the area for biodiversity conservation. Twenty seven mammalian species were identified in the study area. Order Artiodactyla had the highest number of species (11 species), followed by the orders Carnivora (9 species). While, orders Rodentia and Tubulidentata were represented each by one species. Mammal species distribution and abundance in study area vary due to vegetation types and resource availability. Riverine forest had the highest number of species, followed by Savanna grassland. The distribution and utilization of different vegetation communities by mammal species could be explained in terms of seasonal changes. The habitat preferences of the medium and large-sized mammalian species were influenced by seasonal variations in the quality and abundance of forage. It is possible to conclude that food, water, and protection were decisive in determining the distribution of the mammal species in the present study area. Currently, the pressure of intense human activity and improper farming practices pose serious threats to the sustainability of wildlife of the area (S2 Fig.). Thus, study also demonstrates the importance of DWS for broader biodiversity conservation and the requirement for urgent actions that could rehabilitate preservation, management, and conservation of the Sanctuary as well as local mammal species.

Supporting information

S1: Appendix: Supplementary

Acknowledgements: We thank to IDEA-WILD for their material support. We appreciate the management of the Arjo Dhidhessa Sugar Factory. All the supports from the local communities were also sincerely acknowledged.

Authors' contributions: The study data collection was conceptualized and designed by Girma Gizachew, Tadesse Habtamu, Tariku Mekonnen, Tibebu Alemu. Girma Gizachew did the fieldwork, analyzed it, wrote the manuscript, and revised it all. Tadesse Habtamu, Tariku Mekonnen and Tibebu Alemu edited the manuscript and amended the primary document's final version before submitting it for evaluation. The authors participated in the manuscript's writing and approved the final version.

Data Availability: All relevant data is within the paper and it's supporting information files.

Funding: The author(s) received no specific funding for this work.

Competing interests: The authors declare no conflict of interest

Ethics approval: No approval of research ethics committees was required to accomplish the goals of this study.

References

1. IUCN. (2019). IUCN Standards and Petitions Committee. Standards and Petitions Committee, 1(August), 1–60.
2. Zerihun Girma, Yosef Mamo, M. E. (2012). Species composition ,distribution and relative abundance of large mammals in and around Woundo Genet forest patch,Southern Ethiopia.
3. Diriba, G., Tamene, S., & Mengesha, Girma, A. A. (2020). *Diversity of medium and large mammals in the Loka Abaya*. *June*, 9896–9905. https://doi.org/10.1002/ece3.6649
4. Tefera, M. (2011). Wildlife in Ethiopia: Endemic Large Mammals. World Journal of Zoology, 6(2), 108–116.
5. Amare, A. (2015). Wildlife Resources of Ethiopia: Opportunities, Challenges and Future Directions: From Ecotourism Perspective: A Review Paper. *Natural Resources*, *06*(06), 405–422. https://doi.org/10.4236/nr.2015.66039
6. Lavrenchenko, L. A., & Bekele, A. (2017). Diversity and conservation of Ethiopian mammals: What have we learned in 30 years? April 2018.

7. Rabira, G., Tsegaye, G., & Tadesse, H. (2015). The diversity, abundance and habitat association of medium and large-sized mammals of Dati Wolel National Park, Western Ethiopia. International Journal of Biodiversity and Conservation, 7(2), 112–118. https://doi.org/10.5897/ijbc2014.0808
8. Bene, J. K., Bitty, E. A., Bohoussou, K. H., Abedi-, M., Gamys, J., & Soribah, P. A. J. (2013). Current Conservation Status of Large Mammals in Sime Darby Oil Palm Concession in Liberia. *Global Journal of Biology, Agriculture & Health Sciences*, *2*(3), 93–102.
9. Udy, K., Fritsch, M., Meyer, K. M., Grass, I., Hanß, S., Hartig, F., Kneib, T., Kreft, H., Kukunda, C. B., Pe'er, G., Reininghaus, H., Tietjen, B., Tscharntke, T., van Waveren, C. S., & Wiegand, K. (2021). Environmental heterogeneity predicts global species richness patterns better than area. Global Ecology and Biogeography, 30(4), 842–851. https://doi.org/10.1111/geb.13261
10.Gebresenbet, F., Baraki, B., Yirga, G., Sillero-Zubiri, C., & Bauer, H. (2018). A culture of tolerance: Coexisting with large carnivores in the Kafa Highlands, Ethiopia. Oryx, 52(4), 751–760. https://doi.org/10.1017/S0030605316001356
11. Reeder, D. M., Helgen, K. M., & Wilson, D. E. (2019). Global trends and biases in new mammal species discoveries. Global Trends and Biases in New Mammal Species Discoveries. https://doi.org/10.5962/bhl.title.156951
12.Wolf, C., & Ripple, W. J. (2018). Rewilding the world 's large carnivores Subject Category : Subject Areas : Author for correspondence : Royal Society Open Science, 5.
13. Ripple, W. J., Estes, J. A., Beschta, R. L., Wilmers, C. C., Ritchie, E. G., Hebblewhite, M., Berger, J., Elmhagen, B., Letnic, M., Nelson, M. P., Schmitz, O. J., Smith, D. W., Wallach, A. D., & Wirsing, A. J. (2014). Status and ecological effects of the world's largest carnivores. Science, 343(6167). https://doi.org/10.1126/science.1241484
14. Girma, Z., & Worku, Z. (2020). Large Mammal Diversity in Nensebo Forest, Southern Ethiopia. International Journal of Zoology, 2020(December). https://doi.org/10.1155/2020/8819019
15. Mengistu Wale, Abeje Kassie, Getachew Mulualem, Weldemariam Tesfahunegny, A. A. (2017). Wildlife Threats and Their Relative Severity of Eastern Ethiopia Protected Areas. Ecology and Evolutionary Biology, 2(4), 59. https://doi.org/10.11648/j.eeb.20170204.12
16. Qufa, C. A., & Bekele, A. (2019). A preliminary survey of medium and large-sized mammals from Lebu Natural Protected Forest, Southwest Showa, Ethiopia. Ecology and Evolution, 9(21), 12322–12331. https://doi.org/10.1002/ece3.5733
17. Asefa, M., Cao, M., He, Y., Mekonnen, E., Song, X., & Yang, J. (2020). Ethiopian vegetation types, climate and topography. *Plant Diversity*, *42*(4), 302–311. https://doi.org/10.1016/j.pld.2020.04.004
18. Kasso, M., & Bekele, A. (2017). Diversity , Abundance and Distribution of Mammals in Fragmented Remnant Forests around Asella Town, Ethiopia. MAYFEB Journal of Biology, 1(January), 1–12.
19. Belete Tilahun and Melese Merewa (2016). Assessment of large mammals potential in Tululujia Wild life Reserve, South western Ethiopia
20. Ahumada, J., Faresin, C., Gajapersad, K., Hallam, C., Hurtado, J., Martin, E., McWilliam, A., Mugerwa, B., O'Brien, T., Rovero, F., Sheil, D., Spironello, W., Winarni, N. and Andelman, S. (2011). Community structure and diversity of tropical forest mammals: data from a global camera trap network. Philosophical Transactions of the Royal Society

B: Biological Sciences. 366: 2703–2711.
21. Alemu, M. C. (2010). Mammalian Diversity in Borena-Sayint National Park, South Wollo, Ethiopia. 1–74..
22. Dereje Negeri, Tsegaye Gadisa, Tadese Habtamu (2015). The Diversity, Distribution and Relative Abundance of Medium and Large-sized Mammals in Baroye Controlled Hunting Area, Illubabor Zone, Southwest Ethiopia. International Journal of Molecular
23. Geleta Mosissa & Bekele Afework. (2016). Survey of Medium and Large-sized Mammals in Wacha Protected Forest , Western Ethiopia. Scholarly Journal of Agricultural Science, 6(3), 71–79.
24. Gizachew, G., Tefera, Tessema, T. H., & Bekere, Tibebu Alemu Bekere, T. M. G. (2024). Human- common hippo (Hippopotamus amphibius) -conflict in the Dhidhessa Wildlife Sanctuary and its surrounding , Southwestern. PLoS ONE, 1–21. https://doi.org/10.1371/journal.pone.0303647
25. NMA, 2022. National Meteorological Agency, Addis Ababa, Ethiopia.
26. Mamo, Y., Mengesha, G., Fetene, A., Shale, K., & Girma, M. (2016). Statu s of the Swayne ' s Hartebeest , (Alcelaphus buselaphus swaynei) meta-population under land cover changes in Ethiopian Protected Areas. September 2012. https://doi.org/10.5897/IJBC12.024
27. Varman, K. S., & Sukumar, R. (1995). The line transect method for estimating densities of large mammals in a tropical deciduous forest: An evaluation of models and field experiments. Journal of Biosciences, 20(2), 273–287. https://doi.org/10.1007/BF02703274 Evolution and Biodiversity, 5(4):1-9
28. Regassa, R., & Yirga, S. (2013). Distribution , abundance and population status of B urchell ' s zebra (Equus quagga) in Yabello Wildlife. 5(3), 40–49. https://doi.org/10.5897/JENE2012.0352
29. Legese, K., Bekele, A., & Kiros, S. (2019). A Survey of large and medium-sized mammals in Wabe forest fragments, Gurage zone, Ethiopia. International International Journal of Avian & Wildlife Biology, 4(2), 32–38. https://doi.org/10.15406/ijawb.2019.04.00149
30. Maíra Benchimol. (2016). Medium and Large-sized Mammals. Conservation International, April, 207.
31. M.Norton-Griffiths. (1978). Norton-Griffith MN. (1978). Counting Animals (2nd edition). African Wildlife Foundation, Nairobi, Kenya, pp.1-25. In Journal of Chemical Information and Modeling (Vol. 53, Issue 9).
32. Kingdon, J. (2015). Kingdon field guide to African mammalian species-bloomsbury natural history (2nd ed.).
33. Larsen, T. H. (2016). Core Standardized Methods for Rapid Biological Field Assessment. Conservation International, June, 207.
34. Shannon, C. E., & Weaver, W. (1949). The mathematical theory of communication. Chicago, IL: University of Illinois Press. (pp. 3–35).
35.IUCN. (2019).The IUCN Red List of Threatened Species: https://doi.org/10.2305/IUCN.UK.2019-2.RLTS.T41695A147992107.
36. Njoroge, P., Yego, R., Muchane, M., Githiru, M., Njeri, T., & Giani, A. (2009). A Survey of the Large and Medium Sized Mammals of Arawale National Reserve, Kenya. Journal of East African Natural History, 98(1), 119–128. https://doi.org/10.2982/028.098.0108
37. Negeri, D., Gadisa, T., & Habtamu, T. (2015). The Diversity, Distribution and Relative Abundance of Medium and Large-sized Mammals in Baroye Controlled Hunting Area,

Illubabor Zone, Southwest Ethiopia. International Journal of Molecular Evolution and Biodiversity. https://doi.org/10.5376/ijmeb.2015.05.0004

38. Gebrecherkos Woldegeorgis and Tilaye Wube. (2012). A Survey On Mammals Of The Yayu Forest In Southwest Ethiopia. 35(2), 135–138.

39. UNEP-WCMC. (2010). UNEP-WCMC: Review of species/country combinations subject to long-standing import suspensions: Mammal and bird species from Africa. UNEP-WCMC, Cambridge. Pp. 109

40. Tesso, G. J. (2015). Large Mammal Diversity and the Ecology of African Buffalo (Syncerus caffer Sparrman, 1779) in Dhati Wolel National Park, West Ethiopia. November.

41. Indiris Abdu, Gutema Jira and Gatriay Tut (2018).Diversity and Relative Abundance of Medium and Large Mammals in Birbir Protected Forest, Western Ethiopia

42. Du toit, T. and Cumming, D.H. (1999). “Functional significance of ungulate diversity in African savannas and the ecological implications of the spread of pastoralist.”Biodiversity Conservation, 8, 1643‒1661.

43. Legendre, P. and Legendre, L. (2012). Numerical Ecology, 3rd edn. Elsevier, Amsterdam.

44. Erena, M. G. (2021). Assessment of medium and large-sized mammals and their behavioral response toward anthropogenic activities in Jorgo-Wato Protected Forest, Western Ethiopia. *Ecology and Evolution*, *12*(2), 1–13. https://doi.org/10.1002/ece3.8529.

45. O’Kane C, Macdonald D. Seasonal influences on ungulate movement within a fenced South African reserve. J Trop Ecol. 2018;34(3):200–3.

46.Gebo, B., Takele, S., & Shibru, S. (2021). Impacts of habitats and seasons on mammalian diversity and distribution in the Faragosa-Fura landscape, Gamo Zone, Southern Ethiopia. *Geology, Ecology, and Landscapes*, *00*(00), 1–12. https://doi.org/10.1080/24749508.2021.1944798

Table 1. Area of study and transect line

Habitat type	Area (km^2)	Number of sample transects	Length and width of the transects (km)
Riparian forest	17.75	4	5x0.2
Wooded grassland	38.5	10	5x0.2
Savanna grassland	27.2	7	5x0.2
Seasonally flooded grass land	15.11	4	5x0.2
Total	98.56	25	

Table 2: Mammalian species identified in study area (F = Foot prints; S = Seen; P = Photographs; D = Droppings; B = Borrows, * = Community information)

Order	Family	Species	Common name	Local name (Oromifa)	Sign recorded

Primate	Cercopithecidae	*Cercopithecus mittis*	Blue monkey	Qamalee	F,S,P
		Chlorcebus aethiops	Vervet monkey	Qamalee	S,P, F
		Papio anubis	Anubis baboon	Jaldeesa	S,F,P
		Cercopethicus neglectus	DeBrazzes monkey	Chano	S,P
	Colobinae	*Colobus guerza*	Gureza	weenni	S,P,F
Artiodactyla	Suidae	*Phacochoerus africanus*	Warthog	Karkaroo	P,S,D
		Potamochaerus larvatus	Bush pig	Booyye	S,P
		Hylochoerus meinertzhageni	Giant forest hog	Booyye	P
	Hippopotamidae	*Hippopotamus amphibius*	Hippopotamus	Roobii	S,P,F,
	Bovidae	*Syncerus caffer*	African buffalo	Gafarsa	S,P,F,D
		Tragelaphus scriptus	Bush buck	Bosonu	P,S
		Sylvicapra grimmia	Bush Duiker	Quruphe	P,F,D
		Kobus ellipsiprymnus	water buck	Worabbaa	*
		Oreotragus oreotragus	Klipspringer		*
		Redunca redunca	Reedbuck		*
		Ourebia ourebi	Oribi		*
Carnivora	Felidae	*Panthera leo*	Lion	Leenca	F,D,*
		Panthera pardus	Leopard	Qerrensa	P,D
		Leptailurus serval	Serval cat	Iya	*
		Felis caracal	Caracal		*
	Hynaenidae	*Crocuta Crocuta*	Spotted Hyena	Warabessa	P,D
	Mustelidae	*Mellivora capensis*	Honey badger	Amaaqexa	*
	Viverridae	*Civettictis civetta*	African civet	Xirinyi	*
	Canidae	*Canis aureus*	Common jackal	Wangoo	F,P
		Canis mesomelas	Black blacked jackal	Jeedala	*
Rodentia	Hystricidae	*Histrix cristata*	Crested porcupine	Dhaddee	P,S
Tubulidentata	Orycteropodidae	*Orycteropus afer*	Aardvark	Waldigessa	S

Table 3: Relative abundances of mammalian species recorded in the study areas

Species name	Number of individuals recorded		Relative abundance (%)		
	Dry season	Wet season	Dry season	Wet season	Total
Cercopithecus mittis	75	53	6.9	5.7	6.3
Chlorocebus aethiops	142	127	12.9	13.7	13.4
Papio anubis	261	239	23.9	25.8	24.9
Cercopethicus neglectus	28	17	2.56	1.84	2.2
Colobus guerza	87	79	7.96	8.5	8.23
Phacochoerus africanus	13	8	1.19	0.9	1.05
Hylochoerus meinertzhageni	18	13	1.7	1.4	1.6
Potamochaerus larvatus	37	24	3.4	2.6	3
Hippopotamus amphibius	231	133	21.13	14.4	17.8
Syncerus caffer	28	45	2.56	4.9	3.7
Tragelaphus scriptus	32	24	2.93	2.6	2.8
Sylvicapra grimmia	23	12	2.1	1.3	1.7
Kobus ellipsiprymnus	12	6	1.09	0.65	0.9
Oreotragus oreotragus	*	*	*	*	*
Ourebia ourebi	*	*	*	*	*
Redunca redunca	*	*	*	*	*
Panthera leo	12	9	1.09	0.97	1.03
Panthera pardus	8	5	0.73	0.54	0.64
Leptailurus serval	16	27	1.5	2.9	2.2
Crocuta crocuta	11	36	1.01	3.9	2.4
Felis caracal	*	*	*	*	*
Mellivora capensis	9	14	0.8	1.5	1.2
Civettictis civetta	8	5	0.73	0.54	0.64
Canis aureus	12	10	1.09	1.08	1.08
Canis mesomelas	*	*	*	*	*
Histrix cristata	19	17	1.74	1.84	1.8
Orycteropus afer	11	23	1	2.48	1.74
Total	1093	926	100	100	100

Table 4: Occurrence of medium and large sized mammals in the study areas

Species name	Local name	Occurrence category	IUCN category
Cercopithecus mittis	Blue monkey	Frequent	VU
Chlorocebus aethiops	Vervet monkey	Common	LC
Papio Anubis	Anubis baboon	Common	LC
Cercopethicus neglectus	DeBrazzes monkey	Frequent	LC
Colobus guerza	Gureza	Common	LC
Phacochoerus africanus	Warthog	Occasional	LR/LC

Hylochoerus meinertzhageni	Bush pig	Frequent	LR/LC
Potamochaerus larvatus	Giant forest hog	Frequent	LR/LC
Hippopotamus amphibius	Hippopotamus	Common	VU
Syncerus caffer	African buffalo	Occasional	NT
Tragelaphus scriptus	Bush buck	Occasional	LC
Sylvicapra grimmia	Bush Duiker	Occasional	LR/LC
Kobus ellipsiprymnus	water buck	Occasional	LC
Oreotragus oreotragus	Klipspringer	Rare	LC
Redunca redunca	Reedbuck	Rare	LR/cd
Ourebia ourebi	Oribi	Rare	LR/cd
Panthera leo	Lion	Occasional	VU
Panthera pardus	Leopard	Rare	VU
Leptailurus serval	Serval cat	Rare	LC
Crocuta crocuta	Spotted Hyena	Frequent	LC
Hyaena hyaena	Striped hyena	Rare	NT
Mellivora capensis	Honey badger	Occasional	LC
Civettictis civetta	African civet	Occasional	LC
Canis aureus	Common jackal	Occasional	LC
Canis mesomelas	Black blacked jackal	Rare	LC
Histrix cristata	Crested porcupine	Occasional	LC
Orycteropus afer	Aardvark	Occasional	LC

Abbreviations: LR/cd, Lower Risk/ conservation dependent; LR/LC, Lower Risk/ least concern; LC, Least Concern; NT, Near Threatened; VU, Vulnerable

Table 5: Habitat occurrence (%) of large mammals during the wet and dry seasons in the study area

	Number of individuals observed in different habitats (%)			
Season	Riparian forest	Wood land	Savanna grassland	Seasonal flooded grassland
Dry	393 (36)	232 (21.2)	328 (30)	140(12.8)
Wet	339 (36.6)	252 (27.2)	287 (31)	48 (5.18)
Total	732 (36.3)	484 (23.97)	615 (30.5)	188 (9.3)

Table 6: Measuring of biodiversity (Index of diversity) in DWS

	Habitats			
Variables	Riparian forest	Wooded land	Savanna grassland	Seasonal flooded grassland
No. of species	20	19	19	13
No. individuals	732	484	615	188
Dominance_D	0.1482	0.1339	0.1239	0.147
Simpson_1-D	0.8518	0.8661	0.8761	0.853
Shannon_H	2.28	2.346	2.44	2.186
Evenness	0.4889	0.5496	0.6036	0.6849

Manuscript

Click here to access/download;Manuscript;Mammals manuscript with anonymos.docx

The diversity and habitat association of medium and large mammals in the Dhidhessa Wildlife Sanctuary, Southwestern Ethiopia

Abstract

Understanding species diversity and habitat association is the baseline for developing conservation plan. The study aimed to assess diversity, abundance and habitat association of medium and large sized mammals in the Dhidhessa Wildlife Sanctuary, Southwestern Ethiopia. The survey was conducted from December 2022 to June 2023, considering the wet and dry seasons. Stratified random sampling design was applied to stratify the study area in to four (wooded grassland, riparian forest, seasonally flooded grassland, and savanna grassland) strata based on vegetation and habitat type. Line transects survey, sensor camera trapping, indirect and direct evidence methods were used to collect data during wet and dry seasons. Data were analyzed using chi square test and species diversity indexes. Twenty seven mammalian species were recorded for the area. Order Artiodactyla had the highest number of species (eleven) and with the recorded nine species, Order Carnivora follows. While, orders Rodentia and Tubulidentata each represented by one species. *Papio Anubis* (n = 500, 24.9%) was the most abundant species that is followed by *Hippopotamus amphibious* (n = 364, 17.8%) in the present study area in both wet and dry seasons. But *Panthera pardus* (n=13, 0.64%) and *Civettictis civetta* (n=13, 0.64%) were the least abundant species in the present study area. Riverine forest had the highest number of species (n=732, 36.3%) followed by Savanna grassland (n=615, 30.5%). The study revealed that high diversity of species in the area including the rare and threatened species. Therefore, DWS's primary priority should be creating management plans to reestablish the sanctuary as a fully functional sustainable ecosystem and ensuring the social and economic viability of the surrounding community.

Key word: Anthropogenic activities, Dhidhessa Wildlife Sanctua, Diversity, habitat association.

Introduction

Background of the study

Mammals are a remarkable category due to their diversity in species, morphologies, ecologies, and behaviors [1]. The class Mammalia contains 5487 species globally. On the African continent, there are more than 1150 distinct mammalian species [2,3] , with 360 of those species found in eastern Africa [2], and 320 in Ethiopia [4-6] . Fifty-five of the 320 mammalian species known to exist in Ethiopia are endemic [6-7]. Mammalian species support the conservation efforts of other species [8-9], and maintain the ecological balance of terrestrial ecosystems through grazing, predation, and seed dispersal. As a result, they function as umbrella species in the context of ecology [10-11].

Mammals offer significant benefits to humans in terms of the economy, including food and revenue [12]. In contrast, anthropogenic activities that are not appropriate have put mammalian species in danger of extinction both locally and worldwide [13-14]. Nowadays, there are several issues facing mammalian species around the world, especially in developing countries like Ethiopia: habitat degradation due to overgrazing, settlement, hunting, and expanding agriculture [15-17].

There exists a high correlation between the types of habitat occupied by different mammalian species within an ecosystem in many forests [18]. In general, larger ecosystems have more diverse microhabitats and varied vegetation structures. These microhabitats and differing resources are available to species with different feeding and microhabitat requirements, as supported by [3]. In varying seasons and habitat types, mammals are not uniformly dispersed [19]. The main cause of this is that each species has a unique range that is the outcome of the

interaction between the ecological conditions of the present day and the species' evolutionary history. However, many species have similar, though not always identical, distribution patterns. Conversely, individual distribution within a region of easily accessible habitats is indicated by home ranges, territories, and microhabitats [18]. Access to essential resources like food, housing, and water availability determines these. Moreover, changes in the ecosystem's climate, competition, predation, hunting patterns, and habitat all affect the limits of a species' range [20]. As to [7], medium- and large-sized mammals have heightened sensitivity to alterations in their habitat, rendering them dependable markers of the environmental condition. Structurally complex ecosystems may provide more niches and diverse methods of utilizing natural resources, increasing species diversity [21].

According to a study, medium- and large-sized mammals make up more than 60% of Ethiopia's mammal species [22]. The weight of a medium-sized mammal ranged from 2 to 7 kg, whereas that of a large mammal was greater than 7 kg [23]. The diversity of wildlife species found in the Dhidhessa Wildlife Sanctuary is not supported by scientific evidence or official documentation, but numerous oral histories and reports from local informants attest to the abundance of wildlife, particularly mega herbivores and large carnivores, and support the sanctuary's designation from fifty years ago. The country's wildlife resources, including those found in well-established protected areas, have been severely depleted over the past thirty years for a variety of reasons. These include civil war, widespread settlement in the then-wilderness areas, such as the Dhidhesa wildlife sanctuary, widespread settlement during periods of drought, and the unstable and quickly changing central government [7, 24]. Because of the extensive anthropogenic activity around the Dhidhessa River, a greater portion of the wildlife habitats have been destroyed far earlier than the wildlife resources in the area have been documented. This poses a

threat to wildlife habitats,. In addition, the Arjo Dhidhessa sugar plant is the result of the conversion of nearby regions surrounding the sanctuary into automated sugarcane farms [24]. Therefore, the current study was conducted at DWS, one of the wildlife sanctuaries in southwest Ethiopia, where scientific data are essential for studying the diversity and habitat association of medium- and large-sized mammals. These studies serve as the foundation for additional conservation and management strategies.

The study area and methods

Description of the Study Area

The study was conducted in Dhidhessa Wildlife Sanctuary (DWS) and its surrounding areas in southwestern Ethiopia. The Sanctuary is located between $8^{0}30'$ and $8^{0}40'$ N latitude and between $36^{0}22'$ and $36^{0}43'$ E longitude and covers a total area of 1300 km^2. The sanctuary is about 395 km southwest of Addis Ababa (the capital city) and shared among three administrative zones (East Wollega, Buno Bedele, and Jimma), all within Oromia National Regional State (Fig. 1). The altitude within the sanctuary ranges between 1350 and 1050 masl. The sanctuary has two different seasons, dry (November to February) and wet (June to September), with uni-modal rainfall ranging from 648 mm to 2001.8 mm. The sanctuary experienced relatively warm temperatures, with mean annual minimum and maximum temperatures of 12°C and 35°C [25]. In addition to the vast lowland plains along the Dhidhessa river bank, the land form of the sanctuary includes undulating hilly terrain and mountains surrounding the sanctuary from the north and western sides. Habitats in the study area include wooded grassland, riparian forests, seasonal flooded prairies, and extensive savannah grassland. This region was once home to a variety of wildlife species (especially antelopes, mega herbivores, and large carnivores). For the conservation of the habitats and the large mammals therein, the Ethiopian government

designated the DWS in 1970. Before the management system of the sanctuary was well established, for various reasons (including the political instability that spans over three decades and the extensive settlement program for people displaced from the north and eastern parts of the country), part of the then wildlife areas were taken and other parts were heavily encroached. In addition, the extensive plain area adjacent to the sanctuary from the northeastern side was used for the mechanized sugarcane farm to produce raw materials for the state-owned Arjo Dhidhessa Sugar Factory (ADSF). As a result, some evaluative reports show that the DWS prematurely hindered itself from achieving the objectives for which it was established [24].

Methods

A preliminary survey was carried out in February 2021 in the study area to secure information such as the climatic conditions, habitat types, topographic information, level of anthropogenic activities, etc. The Dhidhessa Wildlife Sanctuary (DWS) was characterized by heterogeneous habitats. Based on the vegetation types, the habitat types of the study area were classified into four (riparian forest, savanna grassland, seasonally flooded grassland and wooded grassland) [2,26]. During the preliminary survey, sampled study sites were determined (Table1).

Transect lines counts were used to determine the diversity, and habitat association of medium and large sized mammals. Transect lines was placed on the stratified habitat types in a random fashion and proportional to an area of the habitat type [27]. A total of 25 lines transects, covering about 25% (24.65 Km^2) of the study area. Wildlife trails, footpaths, and other access routes were also used as transect lines. The average length of line transect was 5 km and the width were determined by the vegetation type and season, and ranges between 75m (riverine and tall grass bearing wooded grassland) and 200 m (open grasslands) and each transect ends about a km from the habitat edge [28]. Adjacent transect lines were spaced between one and two Km.

Data collection was conducted once a month for a total of 6 months during both the dry seasons (December, January and February, 2022) and the wet seasons (April, May and June, 2022) to assess the diversity, and habitat association of medium and large sized mammals of DWS. Mammal survey was carried out for three days per season and two times per day 8:00 a.m.to 10:00 a.m. in morning and 4:00 p.m. to 6:00 p.m. late afternoon, when most diurnal medium and large mammals were more active in the study area [29-30]. Each transect line of each habitat type was visited once for a month and at the same period (early morning and evening). During data collection the researcher and 24 assistants person were walking on foot along transect line established and directly count all the individuals of medium and large mammals sighted with their respect species using naked eye and digital camera [31]. Upon detection of animals; habitat types, season of the year, species name and number of individuals observed was record. For identification of mammal species, local expertise and *Kingdon Field Guide to African Mammals* [32] was used.

During the line transect survey, indirect evidences such as Bushnell wildlife camera traps, scats, foot print & hoof and pug marks, hair remains, characteristic burrows, kill remains, etc., were also used to determine the presence/absence of the species and their relative abundance [33]. All basic information of the observed mammals (the species, group size, habitats types, the season, etc.) was recorded.

Data analysis

All data collected during the study period were presented in a table by season and habitat type. The Shannon–Wiener Diversity Index was used [34] to calculate the distribution, abundance, and evenness of species between the wet and dry seasons and within habitat types, and SPSS version 20 software was used to analyze the data. Excel version 2016 was also used to generate the

relative abundance and species diversity indexes using formatted formulas. Moreover, the conservation status of mammals identified from DWS was described as vulnerable and least concerned as per the IUCN Red List [35]. A Chi-Square ($\chi 2$) check, at 0.05 degree of significance might be used to evaluate differences in the abundance of mammal species between habitats and the standard seasonal distinction in the abundance of medium and large-sized mammal species in the examination vicinity.

Results

Species composition and richness

In the study area, during the dry and wet seasons, a total of 27 species of medium- and large-sized animals from five orders and twelve families were identified (Table 2). Of these, 1511 photographs taken with a camera trap over the research periods demonstrated the presence of 11 animal species (S1Fig.). ~~Five orders—Primate, Artiodactyla, Carnivora, Rodentia, and Tubulidentata—were used to group the recorded mammals.~~ With eleven species, the order Artiodactyla has the most of all the orders, followed by the order Carnivora with nine species. Conversely, one species each represented the orders Rodentia and Tubulidentata. With seven species, the Bovidae family was the largest family ~~at the family level~~, followed by the Cercopithecidae family. On the other hand, only one species represented each of the six families. Of the 27 species of medium- and large-sized mammals found in the current study area, five species—the crested porcupine (*Histrix cristata*), honey badger (*Mellivora capensis*), black-blacked jackal (*Canis mesomelas*), common jackal (*Canis aureus*), and aardvark (*Orycteropus afer*)—accounted for 18.5% of the total. The remaining 22 species, or 81.5 %, were large mammals (Table 2).

Species relative abundance and conservation status of mammals

There were significant variations in the seasonal species abundance of medium- and large-sized mammals, with a total of 2019 individuals reported, of which 1093 (54.1%) were observed during the dry season and 926 (45.9%) individuals during the rainy season ($\chi2 = 13.81$, df = 1, $p<0.05$). *Papio anubis* represented (n = 500, 24.9%) was the most abundant species individuals recorded followed by *Hippopotamus amphibious* (n = 364, 17.8%). On the other hand, *Civettictis civetta* (n = 13, 0.64%) and *Panthera pardus* (n = 13, 0.64%) were the two least abundant species (Table 3).

Out of the 27 species of mammals identified in DWS, 11 (40.7%) were considered occasional, 5 (18.5%) frequent, 4 (14.8%) common, and 7 (25.9%) rare. Comparably, Table 4 demonstrates that of the known mammalian species, about 70.4% were categorized as least concern and 14.8% as vulnerable. However, the remaining 14.8% are thought to be conservation-dependent, near threatened, and at lower risk (Table 4).

Habitat association of mammals

In the research area, seasonal variations in the distribution of medium and large mammal species were noted (Table 5). For example, mammals regularly used riparian forests and savanna grasslands in both wet and dry seasons. In contrast, animals used the seasonal flooded grassland (9.3%) less frequently in both seasons (Table 5). There was a noticeable difference in the distribution of indicators linked to mammals' observations in various habitat types during the wet and dry seasons ($\chi2 = 18.87$, df = 3, $p < .05$).

Species diversity and similarity indices

Table 6 shows that compared to other habitats, the Shannon diversity of mammal species was higher in the savanna grassland (H = 2.44) and forest (H = 2.35). Conversely, seasonal flooded

grasslands had the lowest species diversity (H′=2.18).Nonetheless, there was no significant variation in the Shannon-Wiener Index values among the four habitat types. Seasonally flooded grasslands (E = 0.6849) and riparian forests (E = 0.4889) showed the highest and lowest evenness of the mammalian species, respectively. On the other hand, in the riparian forest (D = 0.1482) and the savanna grassland (D = 0.1239), the highest and lowest dominance of mammalian species were observed (S1 table). Additionally, during the dry and wet seasons, the dominance of mammalian species and the Shannon different characteristics record were comparable (S2 table). The research area's maximum species diversity (0.8761) was shown by Simpson's index of diversity.

Discussion

Diversity and relative abundance

The topography, climate, and vegetation types of the current research area have produced a variety of habitats for medium- and large-sized animal species. For example, the 27 species of medium- and large-sized mammals found in the current study area had a significantly higher diversity than the 23 species of mammals found in Arawale National Reserve, Kenya,[36], the 23 species found in the Baroye control hunting area [37], the 22 species found in the fragmented remnant forest near Asella town [18], , a total of 18 species were observed by [2], around the Wondo Genet forest area, 14 species of medium- and large-sized mammals were recorded in Yayu forest by [38], and 12 species of mammals were identified in the Wabe forest's fragments by [29]. A higher level of mammalian diversity in the current research area could have been caused by sufficient vegetation cover and resource availability. Direct observations were also used to identify the majority of the animal species found in the current research area. [23]

revealed that the Watch protected forest produced the same results. The fact that the habitats are so open, maybe as a result of habitat loss and fragmentation, allows for the detection of mammals with the unaided eye. The current study region has a high degree of secretiveness, illusiveness, shyness, and human disturbance, which contributes to the low number of large mammal species that were identified by indirect evidence rather than direct sighting. This outcome was comparable to research conducted by [2] in Loka Abaya National Park and by [29] from Wabe forest fragments.

In contrast to the high diversity of animals (41) documented in Gambella National Park, Ethiopia [39] and 31 species recorded from Dhati Wolel National Park, Ethiopia [40] the results showed comparatively lesser diversity in the study area. The lower diversity of mammals observed in the current study area could be attributed to fragmentation caused by many anthropogenic activities, severe habitat loss, conflict between humans and wildlife, or low level of attitude among local residents toward conservations mammals [24]. Similarly, [41] noted that the demand for wide areas, overexploitation, and habitat fragmentation have led to decline in medium- and large-sized mammals.

The orders Artiodactyla (11 species) and Carnivora (9 species) were the most abundantly seen mammal groups in the current study region. The findings of [40] in Dhati Wolel National Park are consistent with the high number of Artiodactyla in the current research region. With the largest diversity of ungulates on Earth, the African savanna environment has supported multispecies animal production systems for thousands of years [42]. The large proportion of riverine habitats and periodically flooded grasslands that supplied enough grass for grazing during the dry season was probably responsible for the high diversity of herbivores in the current study area. In addition, during the dry season, fires occur in the savanna grassland that is dotted

with Combertum-Terminalia woods. This would enable the high-nutrient new grasses to regenerate, supporting a wide variety of grazers throughout the wet season. Rainfall, fire and herbivory are the prime driving variables in African savanna ecosystems [42]. On the other hand, a large number of different and prolific herbivore species are thought to be responsible for the high diversity of carnivores seen in DWS. Large groups of herbivores, including hippopotamus and African buffalo, were regularly seen throughout the current study, suggesting their high abundance in the region and their ability to serve as a plentiful source of prey for a variety of large carnivore species [43].

Relative abundance

In the current study area, during both the wet and dry seasons, *Papio anubis* was the species with comparatively higher abundance. Nevertheless, the two least common species were *Civettictis civetta* and *Panthera pardus*. Given that the species is suited to eating a wide range of foods and endure various climatic and topographic differences, this may have something to do with its feeding habits. Similar finding was reported by [41] in Birbir protected forest western Ethiopia. In contrast, carnivores were the least common in the study area in terms of both number and distribution. According to respondents, the presence of illegal hunting as a result of widespread livestock predation by carnivores (especially spotted hyena and common jackal) in and around the research area significantly lowers their population. Because of human interference with their cryptic and nocturnal behaviors, it was difficult to document their presence. Similarly, least abundance of carnivores also reported in Dati Wolel National park [7].

Additionally, despite the fact that the area is surrounded by a landscape that is dominated by humans and frequently poses a threat to their survival, many mammals have the ability to transition from diurnal to nocturnal or crepuscular activity. In the riverine forest, however,

nonhuman monkey species were frequently sighted. This could be because nonhuman primates have successfully adapted to human disturbance and changing environments. Similar finding was reported by [44] in the Jorgo-Wato Protected Forest in Western Ethiopia.

Habitat association of mammals

Mammals in the current research region select one habitat over another depending on a variety of factors, including soil types, topography, water availability, and the composition, quality, and availability of feed [16, 23, and 45]. In the research area, riverine forest supported a large number of mammal species during both seasons, followed by savanna grassland. Seasonally flooded grasslands sustained the least amount of diversity. The abundance of food, water, and vegetation cover may be the cause of this high observation rate, but the lack of refuges and vegetation cover may be the reason for the low number of mammal individuals. Similar result was reported by [16, 23, and 41]

Species diversity indices

The richness of species in the various ecosystems varies, as indicated by the species diversity index. The savanna grassland in the current study region, for instance, has the highest species diversity (H′=2.44), while the seasonal flooded grassland has the lowest species diversity (H′=2.18). Likewise, studies by [18] showed that variables like habitat quality, species preferences, availability of food resources, dense forest cover, and water are related to variations in the distribution and abundance of mammal species within different habitats, and that these variables are likely to lead to higher species richness. Our findings go counter to those of the study by [23.46], which indicated that the natural forest had a greater species variety. Human pressure on DWS is one potential source of these variations [24].

Here 1) include implication of the finding, not only sumerizing. 2) what is the specific recommendation based on your finding?

Conclusion and recommendations

The current study provided baseline data regarding the presence and abundance of medium- and large-sized animal species in the DWS, southwestern Ethiopia, as well as information about numerous anthropogenic activities. DWS is unique in that it is home to animals with great conservation value, including large mammals like *Sincerus caffer* and *Hippopotamus amphibibius*, and globally threatened species of mammals like *Panthera leo* and *Panthra pardus*, which together indicate the area for biodiversity conservation. Twenty seven mammalian species were identified in the study area. Order Artiodactyla had the highest number of species (11 species), followed by the orders Carnivora (9 species). While, orders Rodentia and Tubulidentata were represented each by one species. Mammal species distribution and abundance in study area vary due to vegetation types and resource availability. Riverine forest had the highest number of species, followed by Savanna grassland. The distribution and utilization of different vegetation communities by mammal species could be explained in terms of seasonal changes. The habitat preferences of the medium and large-sized mammalian species were influenced by seasonal variations in the quality and abundance of forage. It is possible to conclude that food, water, and protection were decisive in determining the distribution of the mammal species in the present study area. Currently, the pressure of intense human activity and improper farming practices pose serious threats to the sustainability of wildlife of the area (S2 Fig.). Thus, study also demonstrates the importance of DWS for broader biodiversity conservation and the requirement for urgent actions that could rehabilitate preservation, management, and conservation of the Sanctuary as well as local mammal species.

Supporting information

S1: Appendix: Supplementary

Acknowledgements: We thank to IDEA-WILD for their material support. We appreciate the management of the Arjo Dhidhessa Sugar Factory. All the supports from the local communities were also sincerely acknowledged.

Authors' contributions: The study data collection was conceptualized and designed by Girma Gizachew, Tadesse Habtamu, Tariku Mekonnen, Tibebu Alemu. Girma Gizachew did the fieldwork, do you mean data analysis? ~~analyzed it~~, wrote the manuscript, and revised it all. Tadesse Habtamu, Tariku Mekonnen and Tibebu Alemu edited the manuscript and amended the primary document's final version before submitting it for evaluation. The authors participated in the manuscript's writing and approved the final version.

Data Availability: All relevant data is within the paper and it's supporting information files.

Funding: The author(s) received no specific funding for this work.

Competing interests: The authors declare no conflict of interest

Ethics approval: No approval of research ethics committees was required to accomplish the goals of this study.

References

1. IUCN. (2019). IUCN Standards and Petitions Committee. Standards and Petitions Committee, 1(August), 1–60.
2. Zerihun Girma, Yosef Mamo, M. E. (2012). Species composition ,distribution and relative abundance of large mammals in and around Woundo Genet forest patch,Southern Ethiopia.
3. Diriba, G., Tamene, S., & Mengesha, Girma, A. A. (2020). *Diversity of medium and large mammals in the Loka Abaya*. *June*, 9896–9905. https://doi.org/10.1002/ece3.6649
4. Tefera, M. (2011). Wildlife in Ethiopia: Endemic Large Mammals. World Journal of Zoology, 6(2), 108–116.
5. Amare, A. (2015). Wildlife Resources of Ethiopia: Opportunities, Challenges and Future Directions: From Ecotourism Perspective: A Review Paper. *Natural Resources*, *06*(06), 405–422. https://doi.org/10.4236/nr.2015.66039
6. Lavrenchenko, L. A., & Bekele, A. (2017). Diversity and conservation of Ethiopian mammals: What have we learned in 30 years? April 2018.

7. Rabira, G., Tsegaye, G., & Tadesse, H. (2015). The diversity, abundance and habitat association of medium and large-sized mammals of Dati Wolel National Park, Western Ethiopia. International Journal of Biodiversity and Conservation, 7(2), 112–118. https://doi.org/10.5897/ijbc2014.0808

8. Bene, J. K., Bitty, E. A., Bohoussou, K. H., Abedi-, M., Gamys, J., & Soribah, P. A. J. (2013). Current Conservation Status of Large Mammals in Sime Darby Oil Palm Concession in Liberia. *Global Journal of Biology, Agriculture & Health Sciences*, *2*(3), 93–102.

9. Udy, K., Fritsch, M., Meyer, K. M., Grass, I., Hanß, S., Hartig, F., Kneib, T., Kreft, H., Kukunda, C. B., Pe'er, G., Reininghaus, H., Tietjen, B., Tscharntke, T., van Waveren, C. S., & Wiegand, K. (2021). Environmental heterogeneity predicts global species richness patterns better than area. Global Ecology and Biogeography, 30(4), 842–851. https://doi.org/10.1111/geb.13261

10.Gebresenbet, F., Baraki, B., Yirga, G., Sillero-Zubiri, C., & Bauer, H. (2018). A culture of tolerance: Coexisting with large carnivores in the Kafa Highlands, Ethiopia. Oryx, 52(4), 751–760. https://doi.org/10.1017/S0030605316001356

11. Reeder, D. M., Helgen, K. M., & Wilson, D. E. (2019). Global trends and biases in new mammal species discoveries. Global Trends and Biases in New Mammal Species Discoveries. https://doi.org/10.5962/bhl.title.156951

12.Wolf, C., & Ripple, W. J. (2018). Rewilding the world 's large carnivores Subject Category : Subject Areas : Author for correspondence : Royal Society Open Science, 5.

13. Ripple, W. J., Estes, J. A., Beschta, R. L., Wilmers, C. C., Ritchie, E. G., Hebblewhite, M., Berger, J., Elmhagen, B., Letnic, M., Nelson, M. P., Schmitz, O. J., Smith, D. W., Wallach, A. D., & Wirsing, A. J. (2014). Status and ecological effects of the world's largest carnivores. Science, 343(6167). https://doi.org/10.1126/science.1241484

14. Girma, Z., & Worku, Z. (2020). Large Mammal Diversity in Nensebo Forest, Southern Ethiopia. International Journal of Zoology, 2020(December). https://doi.org/10.1155/2020/8819019

15. Mengistu Wale, Abeje Kassie, Getachew Mulualem, Weldemariam Tesfahunegny, A. A. (2017). Wildlife Threats and Their Relative Severity of Eastern Ethiopia Protected Areas. Ecology and Evolutionary Biology, 2(4), 59. https://doi.org/10.11648/j.eeb.20170204.12

16. Qufa, C. A., & Bekele, A. (2019). A preliminary survey of medium and large-sized mammals from Lebu Natural Protected Forest, Southwest Showa, Ethiopia. Ecology and Evolution, 9(21), 12322–12331. https://doi.org/10.1002/ece3.5733

17. Asefa, M., Cao, M., He, Y., Mekonnen, E., Song, X., & Yang, J. (2020). Ethiopian vegetation types, climate and topography. *Plant Diversity*, *42*(4), 302–311. https://doi.org/10.1016/j.pld.2020.04.004

18. Kasso, M., & Bekele, A. (2017). Diversity , Abundance and Distribution of Mammals in Fragmented Remnant Forests around Asella Town, Ethiopia. MAYFEB Journal of Biology, 1(January), 1–12.

19. Belete Tilahun and Melese Merewa (2016). Assessment of large mammals potential in Tulului jia Wild life Reserve, South western Ethiopia

20. Ahumada, J., Faresin, C., Gajapersad, K., Hallam, C., Hurtado, J., Martin, E., McWilliam, A., Mugerwa, B., O'Brien, T., Rovero, F., Sheil, D., Spironello, W., Winarni, N. and Andelman, S. (2011). Community structure and diversity of tropical forest mammals: data from a global camera trap network. Philosophical Transactions of the Royal Society

B: Biological Sciences. 366: 2703–2711.

21. Alemu, M. C. (2010). Mammalian Diversity in Borena-Sayint National Park, South Wollo, Ethiopia. 1–74..
22. Dereje Negeri, Tsegaye Gadisa, Tadese Habtamu (2015). The Diversity, Distribution and Relative Abundance of Medium and Large-sized Mammals in Baroye Controlled Hunting Area, Illubabor Zone, Southwest Ethiopia. International Journal of Molecular
23. Geleta Mosissa & Bekele Afework. (2016). Survey of Medium and Large-sized Mammals in Wacha Protected Forest , Western Ethiopia. Scholarly Journal of Agricultural Science, 6(3), 71–79.
24. Gizachew, G., Tefera, Tessema, T. H., & Bekere, Tibebu Alemu Bekere, T. M. G. (2024). Human- common hippo (Hippopotamus amphibius) -conflict in the Dhidhessa Wildlife Sanctuary and its surrounding , Southwestern. PLoS ONE, 1–21. https://doi.org/10.1371/journal.pone.0303647
25. NMA, 2022. National Meteorological Agency, Addis Ababa, Ethiopia.
26. Mamo, Y., Mengesha, G., Fetene, A., Shale, K., & Girma, M. (2016). Statu s of the Swayne ' s Hartebeest , (Alcelaphus buselaphus swaynei) meta-population under land cover changes in Ethiopian Protected Areas. September 2012. https://doi.org/10.5897/IJBC12.024
27. Varman, K. S., & Sukumar, R. (1995). The line transect method for estimating densities of large mammals in a tropical deciduous forest: An evaluation of models and field experiments. Journal of Biosciences, 20(2), 273–287. https://doi.org/10.1007/BF02703274 Evolution and Biodiversity, 5(4):1-9
28. Regassa, R., & Yirga, S. (2013). Distribution , abundance and population status of B urchell ' s zebra (Equus quagga) in Yabello Wildlife. 5(3), 40–49. https://doi.org/10.5897/JENE2012.0352
29. Legese, K., Bekele, A., & Kiros, S. (2019). A Survey of large and medium-sized mammals in Wabe forest fragments, Gurage zone, Ethiopia. International International Journal of Avian & Wildlife Biology, 4(2), 32–38. https://doi.org/10.15406/ijawb.2019.04.00149
30. Maíra Benchimol. (2016). Medium and Large-sized Mammals. Conservation International, April, 207.
31. M.Norton-Griffiths. (1978). Norton-Griffith MN. (1978). Counting Animals (2nd edition). African Wildlife Foundation, Nairobi, Kenya, pp.1-25. In Journal of Chemical Information and Modeling (Vol. 53, Issue 9).
32. Kingdon, J. (2015). Kingdon field guide to African mammalian species-bloomsbury natural history (2nd ed.).
33. Larsen, T. H. (2016). Core Standardized Methods for Rapid Biological Field Assessment. Conservation International, June, 207.
34. Shannon, C. E., & Weaver, W. (1949). The mathematical theory of communication. Chicago, IL: University of Illinois Press. (pp. 3–35).
35.IUCN. (2019).The IUCN Red List of Threatened Species: https://doi.org/10.2305/IUCN.UK.2019-2.RLTS.T41695A147992107.
36. Njoroge, P., Yego, R., Muchane, M., Githiru, M., Njeri, T., & Giani, A. (2009). A Survey of the Large and Medium Sized Mammals of Arawale National Reserve, Kenya. Journal of East African Natural History, 98(1), 119–128. https://doi.org/10.2982/028.098.0108
37. Negeri, D., Gadisa, T., & Habtamu, T. (2015). The Diversity, Distribution and Relative Abundance of Medium and Large-sized Mammals in Baroye Controlled Hunting Area,

Illubabor Zone, Southwest Ethiopia. International Journal of Molecular Evolution and Biodiversity. https://doi.org/10.5376/ijmeb.2015.05.0004

38. Gebrecherkos Woldegeorgis and Tilaye Wube. (2012). A Survey On Mammals Of The Yayu Forest In Southwest Ethiopia. 35(2), 135–138.

39. UNEP-WCMC. (2010). UNEP-WCMC: Review of species/country combinations subject to long-standing import suspensions: Mammal and bird species from Africa. UNEP-WCMC, Cambridge. Pp. 109

40. Tesso, G. J. (2015). Large Mammal Diversity and the Ecology of African Buffalo (Syncerus caffer Sparrman, 1779) in Dhati Wolel National Park, West Ethiopia. November.

41. Indiris Abdu, Gutema Jira and Gatriay Tut (2018).Diversity and Relative Abundance of Medium and Large Mammals in Birbir Protected Forest, Western Ethiopia

42. Du toit, T. and Cumming, D.H. (1999). “Functional significance of ungulate diversity in African savannas and the ecological implications of the spread of pastoralist.”Biodiversity Conservation, 8, 1643‒1661.

43. Legendre, P. and Legendre, L. (2012). Numerical Ecology, 3rd edn. Elsevier, Amsterdam.

44. Erena, M. G. (2021). Assessment of medium and large-sized mammals and their behavioral response toward anthropogenic activities in Jorgo-Wato Protected Forest, Western Ethiopia. *Ecology and Evolution*, *12*(2), 1–13. https://doi.org/10.1002/ece3.8529.

45. O’Kane C, Macdonald D. Seasonal influences on ungulate movement within a fenced South African reserve. J Trop Ecol. 2018;34(3):200–3.

46.Gebo, B., Takele, S., & Shibru, S. (2021). Impacts of habitats and seasons on mammalian diversity and distribution in the Faragosa-Fura landscape, Gamo Zone, Southern Ethiopia. *Geology, Ecology, and Landscapes*, *00*(00), 1–12. https://doi.org/10.1080/24749508.2021.1944798

Table 1. Area of study and transect line

Habitat type	Area (km^2)	Number of sample transects	Length and width of the transects (km)
Riparian forest	17.75	4	5x0.2
Wooded grassland	38.5	10	5x0.2
Savanna grassland	27.2	7	5x0.2
Seasonally flooded grass land	15.11	4	5x0.2
Total	98.56	25	

Table 2: Mammalian species identified in study area (F = Foot prints; S = Seen; P = Photographs; D = Droppings; B = Borrows, * = Community information)

Order	Family	Species	Common name	Local name (Oromifa)	Sign recorded

Primate	Cercopithecidae	*Cercopithecus mittis*	Blue monkey	Qamalee	F,S,P
		Chlorcebus aethiops	Vervet monkey	Qamalee	S,P, F
		Papio anubis	Anubis baboon	Jaldeesa	S,F,P
		Cercopethicus neglectus	DeBrazzes monkey	Chano	S,P
	Colobinae	*Colobus guerza*	Gureza	weenni	S,P,F
Artiodactyla	Suidae	*Phacochoerus africanus*	Warthog	Karkaroo	P,S,D
		Potamochaerus larvatus	Bush pig	Booyye	S,P
		Hylochoerus meinertzhageni	Giant forest hog	Booyye	P
	Hippopotamidae	*Hippopotamus amphibius*	Hippopotamus	Roobii	S,P,F,
	Bovidae	*Syncerus caffer*	African buffalo	Gafarsa	S,P,F,D
		Tragelaphus scriptus	Bush buck	Bosonu	P,S
		Sylvicapra grimmia	Bush Duiker	Quruphe	P,F,D
		Kobus ellipsiprymnus	water buck	Worabbaa	*
		Oreotragus oreotragus	Klipspringer		*
		Redunca redunca	Reedbuck		*
		Ourebia ourebi	Oribi		*
Carnivora	Felidae	*Panthera leo*	Lion	Leenca	F,D,*
		Panthera pardus	Leopard	Qerrensa	P,D
		Leptailurus serval	Serval cat	Iya	*
		Felis caracal	Caracal		*
	Hynaenidae	*Crocuta Crocuta*	Spotted Hyena	Warabessa	P,D
	Mustelidae	*Mellivora capensis*	Honey badger	Amaaqexa	*
	Viverridae	*Civettictis civetta*	African civet	Xirinyi	*
	Canidae	*Canis aureus*	Common jackal	Wangoo	F,P
		Canis mesomelas	Black blacked jackal	Jeedala	*
Rodentia	Hystricidae	*Histrix cristata*	Crested porcupine	Dhaddee	P,S
Tubulidentata	Orycteropodidae	*Orycteropus afer*	Aardvark	Waldigessa	S

Table 3: Relative abundances of mammalian species recorded in the study areas

Species name	Number of individuals recorded		Relative abundance (%)		
	Dry season	Wet season	Dry season	Wet season	Total
Cercopithecus mittis	75	53	6.9	5.7	6.3
Chlorocebus aethiops	142	127	12.9	13.7	13.4
Papio anubis	261	239	23.9	25.8	24.9
Cercopethicus neglectus	28	17	2.56	1.84	2.2
Colobus guerza	87	79	7.96	8.5	8.23
Phacochoerus africanus	13	8	1.19	0.9	1.05
Hylochoerus meinertzhageni	18	13	1.7	1.4	1.6
Potamochaerus larvatus	37	24	3.4	2.6	3
Hippopotamus amphibius	231	133	21.13	14.4	17.8
Syncerus caffer	28	45	2.56	4.9	3.7
Tragelaphus scriptus	32	24	2.93	2.6	2.8
Sylvicapra grimmia	23	12	2.1	1.3	1.7
Kobus ellipsiprymnus	12	6	1.09	0.65	0.9
Oreotragus oreotragus	*	*	*	*	*
Ourebia ourebi	*	*	*	*	*
Redunca redunca	*	*	*	*	*
Panthera leo	12	9	1.09	0.97	1.03
Panthera pardus	8	5	0.73	0.54	0.64
Leptailurus serval	16	27	1.5	2.9	2.2
Crocuta crocuta	11	36	1.01	3.9	2.4
Felis caracal	*	*	*	*	*
Mellivora capensis	9	14	0.8	1.5	1.2
Civettictis civetta	8	5	0.73	0.54	0.64
Canis aureus	12	10	1.09	1.08	1.08
Canis mesomelas	*	*	*	*	*
Histrix cristata	19	17	1.74	1.84	1.8
Orycteropus afer	11	23	1	2.48	1.74
Total	1093	926	100	100	100

Table 4: Occurrence of medium and large sized mammals in the study areas

Species name	Local name	Occurrence category	IUCN category
Cercopithecus mittis	Blue monkey	Frequent	VU
Chlorocebus aethiops	Vervet monkey	Common	LC
Papio Anubis	Anubis baboon	Common	LC
Cercopethicus neglectus	DeBrazzes monkey	Frequent	LC
Colobus guerza	Gureza	Common	LC
Phacochoerus africanus	Warthog	Occasional	LR/LC

Hylochoerus meinertzhageni	Bush pig	Frequent	LR/LC
Potamochaerus larvatus	Giant forest hog	Frequent	LR/LC
Hippopotamus amphibius	Hippopotamus	Common	VU
Syncerus caffer	African buffalo	Occasional	NT
Tragelaphus scriptus	Bush buck	Occasional	LC
Sylvicapra grimmia	Bush Duiker	Occasional	LR/LC
Kobus ellipsiprymnus	water buck	Occasional	LC
Oreotragus oreotragus	Klipspringer	Rare	LC
Redunca redunca	Reedbuck	Rare	LR/cd
Ourebia ourebi	Oribi	Rare	LR/cd
Panthera leo	Lion	Occasional	VU
Panthera pardus	Leopard	Rare	VU
Leptailurus serval	Serval cat	Rare	LC
Crocuta crocuta	Spotted Hyena	Frequent	LC
Hyaena hyaena	Striped hyena	Rare	NT
Mellivora capensis	Honey badger	Occasional	LC
Civettictis civetta	African civet	Occasional	LC
Canis aureus	Common jackal	Occasional	LC
Canis mesomelas	Black blacked jackal	Rare	LC
Histrix cristata	Crested porcupine	Occasional	LC
Orycteropus afer	Aardvark	Occasional	LC

Abbreviations: LR/cd, Lower Risk/ conservation dependent; LR/LC, Lower Risk/ least concern; LC, Least Concern; NT, Near Threatened; VU, Vulnerable

Table 5: Habitat occurrence (%) of large mammals during the wet and dry seasons in the study area

Number of individuals observed in different habitats (%)				
Season	Riparian forest	Wood land	Savanna grassland	Seasonal flooded grassland
Dry	393 (36)	232 (21.2)	328 (30)	140(12.8)
Wet	339 (36.6)	252 (27.2)	287 (31)	48 (5.18)
Total	732 (36.3)	484 (23.97)	615 (30.5)	188 (9.3)

Table 6: Measuring of biodiversity (Index of diversity) in DWS

Habitats				
Variables	Riparian forest	Wooded land	Savanna grassland	Seasonal flooded grassland
No. of species	20	19	19	13
No. individuals	732	484	615	188
Dominance_D	0.1482	0.1339	0.1239	0.147
Simpson_1-D	0.8518	0.8661	0.8761	0.853
Shannon_H	2.28	2.346	2.44	2.186
Evenness	0.4889	0.5496	0.6036	0.6849

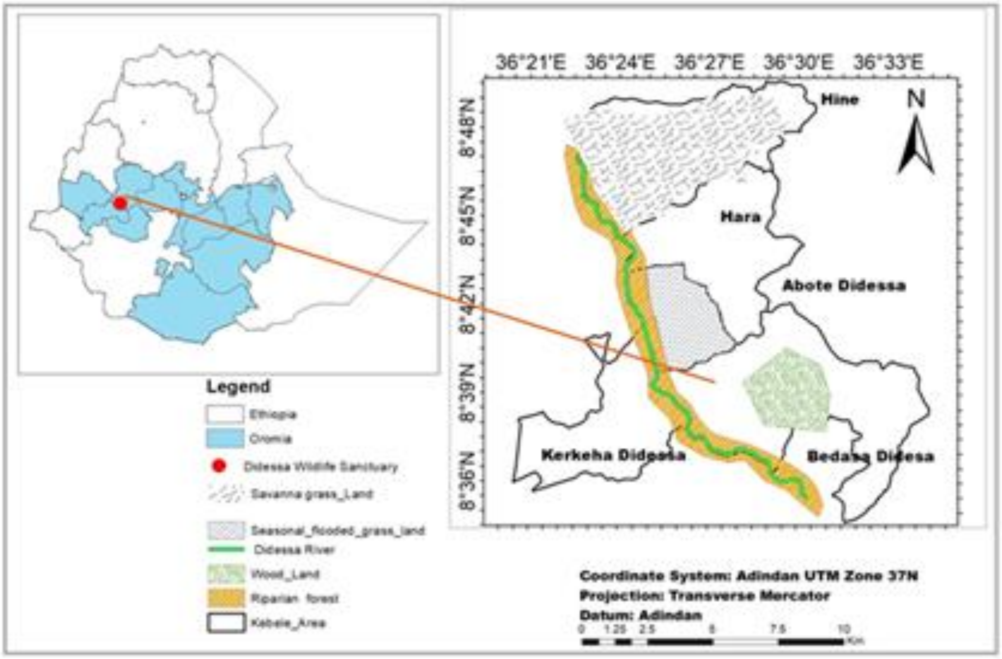

Figure 1: Map of study area (Our team used shape files provided by the Ethiopian Mapping Agency to produce the map. The agency's official website offers free access to these shape files (http://www.ema.gov.et).

Click here to access/download
Supporting Information
Supplementary.docx
