# Supplementary material for: The diversity and habitat association of medium and large mammals in the Dhidhessa Wildlife Sanctuary, Southwestern Ethiopia
Source: PLoS One. 2025 Mar 21;20(3):e0317441. doi: 10.1371/journal.pone.0317441 (PMC11927904; doi:10.1371/journal.pone.0317441)
Supplement: Attachment — Submitted filename: PONE-D-24-30486_reviewer commented manuscript.docx [file pone.0317441.s002.docx]

PLOS ONE

The diversity and habitat association of medium and large mammals in the Dhidhessa Wildlife Sanctuary, Southwestern Ethiopia

--Manuscript Draft--

| Manuscript Number: | PONE-D-24-30486 |
| --- | --- |
| Article Type: | Research Article |
| Full Title: | The diversity and habitat association of medium and large mammals in the Dhidhessa Wildlife Sanctuary, Southwestern Ethiopia |
| Short Title: | Diversity and habitat association of medium and large mammals in the Dhidhessa Wildlife Sanctuary |
| Corresponding Author: | Girma Gizachew Tefera, MSC  Jimma University College of Agriculture and Veterinary Medicine Jimma, ETHIOPIA |
| Keywords: | Anthropogenic activities, Dhidhessa Wildlife Sanctuaryy, Diversity , Habitat association. |
| Abstract: | Understanding species diversity and habitat association is the baseline for developing conservation plan. The study aimed to assess diversity, abundance and habitat association of medium and large sized mammals in the Dhidhessa Wildlife Sanctuary, Southwestern Ethiopia. The survey was conducted from December 2022 to June 2023, considering the wet and dry seasons. Stratified random sampling design was applied to stratify the study area in to four (wooded grassland, riparian forest, seasonally flooded grassland, and savanna grassland) strata based on vegetation and habitat type. Line transects survey, sensor camera trapping, indirect and direct evidence methods were used to collect data during wet and dry seasons. Data were analyzed using chi square test and species diversity indexes. Twenty seven mammalian species were recorded for the area. Order Artiodactyla had the highest number of species (eleven) and with the recorded nine species, Order Carnivora follows. While, orders Rodentia and Tubulidentata each represented by one species. Papio Anubis (n = 500, 24.9%) was the most abundant species that is followed by Hippopotamus amphibious (n = 364, 17.8%) in the present study area in both wet and dry seasons. But Panthera pardus (n=13, 0.64%) and Civettictis civetta (n=13, 0.64%) were the least abundant species in the present study area. Riverine forest had the highest number of species (n=732, 36.3%) followed by Savanna grassland (n=615, 30.5%). The study revealed that high diversity of species in the area including the rare and threatened species.  Therefore, DWS's primary priority should be creating management plans to reestablish the sanctuary as a fully functional sustainable ecosystem and ensuring the social and economic viability of the surrounding community. |
| Order of Authors: | Girma Gizachew Tefera, PhD Candidate |
|  | Tadesse Habtamu Tessema, Dr |
|  | Tibebu Alemu Bekere, Dr |
|  | Tariku Mekonnen Gutema, Dr |
| Additional Information: |  |
| Question | Response |
| Financial Disclosure  Enter a financial disclosure statement that describes the sources of funding for the work included in this submission. Review the [submission guidelines](http://journals.plos.org/plosone/s/submission-guidelines#loc-financial-disclosure-statement) for detailed requirements. View published research articles from [*PLOS ONE*](https://journals.plos.org/plosone/) for specific examples. | The author(s) received no specific funding for this work. |

| This statement is required for submission and will appear in the published article if the submission is accepted. Please make sure it is accurate. |  |
| --- | --- |
| Funded studies  Enter a statement with the following details:   - Initials of the authors who received each award - Grant numbers awarded to each author - The full name of each funder - URL of each funder website - Did the sponsors or funders play any role in the study design, data collection and analysis, decision to publish, or preparation of the manuscript? |  |
| Did you receive funding for this work? |  |
| Competing Interests  Use the instructions below to enter a competing interest statement for this submission. On behalf of all authors, disclose any [competing interests](http://journals.plos.org/plosone/s/competing-interests) that could be perceived to bias this work—acknowledging all financial support and any other relevant financial or non- financial competing interests.  This statement is required for submission and will appear in the published article if the submission is accepted. Please make sure it is accurate and that any funding sources listed in your Funding Information later in the submission form are also declared in your Financial Disclosure statement.  View published research articles from  [*PLOS ONE*](http://journals.plos.org/plosone/) for specific examples. | The authors declare no conflict of interest |

| NO authors have competing interests  Enter: *The authors have declared that no competing interests exist.*  Authors with competing interests  Enter competing interest details beginning with this statement:  *I have read the journal's policy and the authors of this manuscript have the following competing interests: [insert competing interests here]* |  |
| --- | --- |
| * typeset |  |
| Ethics Statement  Enter an ethics statement for this submission. This statement is required if the study involved:   - Human participants - Human specimens or tissue - Vertebrate animals or cephalopods - Vertebrate embryos or tissues - Field research   Write "N/A" if the submission does not require an ethics statement.  General guidance is provided below. Consult the [submission guidelines](http://journals.plos.org/plosone/s/submission-guidelines#loc-guidelines-for-specific-study-types) for detailed instructions. Make sure that all information entered here is included in the Methods section of the manuscript. | Jimma Univesristy |

| Format for specific study types  Human Subject Research (involving human participants and/or tissue)   - Give the name of the institutional review board or ethics committee that approved the study - Include the approval number and/or a statement indicating approval of this research - Indicate the form of consent obtained (written/oral) or the reason that consent was not obtained (e.g. the data were analyzed anonymously)   Animal Research (involving vertebrate animals, embryos or tissues)   - Provide the name of the Institutional Animal Care and Use Committee (IACUC) or other relevant ethics board that reviewed the study protocol, and indicate whether they approved this research or granted a formal waiver of ethical approval - Include an approval number if one was obtained - If the study involved *non-human primates*, add *additional details* about animal welfare and steps taken to ameliorate suffering - If anesthesia, euthanasia, or any kind of animal sacrifice is part of the study, include briefly which substances and/or methods were applied   Field Research  Include the following details if this study involves the collection of plant, animal, or other materials from a natural setting:   - Field permit number - Name of the institution or relevant body that granted permission |  |
| --- | --- |
|  |  |
| Data Availability  Authors are required to make all data underlying the findings described fully available, without restriction, and from the time of publication. PLOS allows rare exceptions to address legal and ethical concerns. See the [PLOS Data Policy](http://journals.plos.org/plosone/s/data-availability) and [FAQ](http://journals.plos.org/plosone/s/data-availability#loc-faqs-for-data-policy) for detailed information. | Yes - all data are fully available without restriction |

| A Data Availability Statement describing where the data can be found is required at submission. Your answers to this question constitute the Data Availability Statement and will be published in the article, if accepted. |  |
| --- | --- |
| Important: Stating ‘data available on request from the author’ is not sufficient. If your data are only available upon request, select ‘No’ for the first question and explain your exceptional situation in the text box. |  |
| Do the authors confirm that all data underlying the findings described in their manuscript are fully available without restriction? |  |
| Describe where the data may be found in full sentences. If you are copying our sample text, replace any instances of XXX with the appropriate details.   - If the data are held or will be held in a public repository, include URLs, accession numbers or DOIs. If this information will only be available after acceptance, indicate this by ticking the box below. For example: *All XXX files are available from the XXX database (accession number(s) XXX, XXX.*). - If the data are all contained within the manuscript and/or Supporting Information files, enter the following: *All relevant data are within the manuscript and its Supporting Information files.* - If neither of these applies but you are able to provide details of access elsewhere, with or without limitations, please do so. For example:   *Data cannot be shared publicly because of [XXX]. Data are available from the XXX Institutional Data Access / Ethics Committee (contact via XXX) for researchers who meet the criteria for access to confidential data.*  *The data underlying the results presented in the study are available from (include the name of the third party* | All relevant data is within the paper and it’s supporting information files |

| *and contact information or URL).*   - This text is appropriate if the data are owned by a third party and authors do not have permission to share the data.   * typeset |  |
| --- | --- |
| Additional data availability information: | Tick here if the URLs/accession numbers/DOIs will be available only after acceptance of the manuscript for publication so that we can ensure their inclusion before publication.; Tick here if your circumstances are not covered by the questions above and you need the journal’s help to make your data available. |

<https://www2.cloud.editorialmanager.com/pone/download.aspx?id=37125486&guid=8c6d2b89-2d89-4d16-b11f-8d17b544dcdd&scheme=1>Manuscript	[Click here to access/download;Manuscript;Mammals manuscript.docx](https://www2.cloud.editorialmanager.com/pone/download.aspx?id=37125486&guid=8c6d2b89-2d89-4d16-b11f-8d17b544dcdd&scheme=1)

1

1 **The diversity and habitat association of medium and large mammals in the Dhidhessa Wildlife**
2 **Sanctuary, Southwestern Ethiopia**

3

4

5 Girma Gizachew Tefera^1^, Tadesse Habtamu Tessema^2^ , Tibebu Alemu Bekere^3^, Tariku
6 Mekonnen Gutema^4^

7

8 ^1^ Department of Natural Resource Management, Colleague of Agriculture and Veterinary
9 Medicine, Jimma University, Jimma, Ethiopia.
10 ^2^ Department of Biology, College of computational Sciences, Jimma University, Jimma, Ethiopia
11 ^3^ Department of Natural Resource Management, Colleague of Agriculture and Veterinary
12 Medicine, Jimma University, Jimma, Ethiopia.
13 ^4^ Department of Natural Resource Management, Colleague of Agriculture and Veterinary
14 Medicine, Jimma University, Jimma, Ethiopia.

15

16

17	Corresponding author: [kennaa20047@gmail.com](mailto:kennaa20047@gmail.com)

| 18 | Tel: | +251917550062 |
| --- | --- | --- |
| 19Short title? |  |  |
| 20 |  |  |
| 21 |  |  |
| 22 |  |  |
| 23 |  |  |
| 24 |  |  |
| 25 |  |  |
| 26 |  |  |
| 27 |  |  |
| 28 |  |  |

### 29 Abstract

30 Understanding species diversity and habitat association is the baseline for developing
31 conservation plan. The study aimed to assess diversity, abundance, and habitat association of
32 medium- and large-sized mammals in the Dhidhessa Wildlife Sanctuary, Southwestern Ethiopia.
33 The survey was conducted from December 2022 to June 2023, considering the wet and dry
34 seasons. A stratified random sampling design was applied to stratify the study area into four
35 (wooded grassland, riparian forest, seasonally flooded grassland, and savanna grassland) strata
36 based on vegetation and habitat type. Line transect survey, sensor camera trapping, and indirect and
37 direct evidence methods were used to collect data during wet and dry seasons. Data were
38 analyzed using the chi square test and species diversity indexes. Twenty-seven mammalian species
39 were recorded for the area. Order Artiodactyla, which had the highest number of species (n = 11)followed by the Order Carnivora (n = 9). While, orders Rodentia and
40 Tubulidentata, each represented by one species. *Papio anubis* (n = 500, 24.9%), were the most
41 abundant species, followed by *Hippopotamus amphibious* (n = 364, 17.8%) in the present
42 study area in both wet and dry seasons. But *Panthera pardus* (n = 13, 0.64%) and *Civettictis*
43 *civetta* (n = 13, 0.64%) were the least abundant species in the present study area. Riverine forest
44 had the highest number of species (n = 732, 36.3%) followed by savanna grassland (n = 615,
45 30.5%). The study revealed a high diversity of species in the area, including the rare and
46 threatened species. Therefore, DWS's primary priority should be creating management plans to
47 re-establish the sanctuary as a fully functional, sustainable ecosystem and ensuring the social and
48 economic viability of the surrounding community.
49 **Keywords:** Anthropogenic activities, Dhidhessa Wildlife Sanctuary, Diversity, Habitat association.

51

### 52 Introduction

53 **Background of the study**
54 Mammals are a remarkable category due to their diversity in species, morphologies, ecologies,
55 and behaviors [1]. The class Mammalia contains 5487 species globally. On the African
56 continent, there are more than 1150 distinct mammalian species [2,3] , with 360 of those species
57 found in eastern Africa [2], and 320 in Ethiopia [4-6]. Fifty-five of the mammalian species
58 known to exist in Ethiopia are endemic [6-7]. Mammalian species support the conservation
59 efforts of other species [8-9], and maintain the ecological balance of terrestrial ecosystems
60 through grazing, predation, and seed dispersal. As a result, they function as umbrella species in
61 the context of ecology [10-11].
62 Mammals offer significant benefits to humans in terms of the economy, including food and
63 revenue [12]. In contrast, anthropogenic activities that are not appropriate have put mammalian
64 species in danger of extinction both locally and worldwide [13-14]. Nowadays, there are several
65 issues facing mammalian species around the world, especially in developing countries like
66 Ethiopia: habitat degradation due to overgrazing, settlement, hunting, and expanding agriculture
67 [15-17].
68 There exists a high correlation between the types of habitat occupied by different mammalian
69 species within an ecosystem in many forests [18]. In general, larger ecosystems have more
70 diverse microhabitats and varied vegetation structures. These microhabitats and differing
71 resources are available to species with different feeding and microhabitat requirements, as
72 supported by [3]. In varying seasons and habitat types, mammals are not uniformly dispersed
73 [19]. The main cause of this is that each species has a unique range that is the outcome of the
74 interaction between the ecological conditions of the present day and the species' evolutionary
75 history. However, many species have similar, though not always identical, distribution patterns.
76 Conversely, individual distribution within a region of easily accessible habitats is indicated by
77 home ranges, territories, and microhabitats [18]. Access to essential resources like food, housing,
78 and water availability determines these. Moreover, changes in the ecosystem's climate,
79 competition, predation, hunting patterns, and habitat all affect the limits of a species' range [20].
80 As to [7], medium- and large-sized mammals have heightened sensitivity to alterations in their
81 habitat, rendering them dependable markers of the environmental condition. Structurally
82 complex ecosystems may provide more niches and diverse methods of utilizing natural
83 resources, increasing species diversity [21].
84 According to a study, medium- and large-sized mammals make up more than 60% of Ethiopia's
85 mammal species [22]. The weight of a medium-sized mammal ranged from 2 to 7 kg, whereas
86 that of a large mammal was greater than 7 kg [23]. The diversity of wildlife species found in the
87 Dhidhessa Wildlife Sanctuary is not supported by scientific evidence or official documentation,
88 but numerous oral histories and reports from local informants attest to the abundance of wildlife,
89 particularly mega herbivores and large carnivores, and support the sanctuary's designation from
90 fifty years ago. The country's wildlife resources, including those found in well-established
91 protected areas, have been severely depleted over the past thirty years for a variety of reasons.
92 These include civil war, widespread settlement in the then-wilderness areas, such as the
93 Dhidhesa wildlife sanctuary, widespread settlement during periods of drought, and the unstable
94 and quickly changing central government [7, 24]. Because of the extensive anthropogenic
95 activity around the Dhidhessa River, a greater portion of the wildlife habitats have been
96 destroyed far earlier than the wildlife resources in the area have been documented. This poses a
97 threat to wildlife habitats,. In addition, the Arjo Dhidhessa sugar plant is the result of the
98 conversion of nearby regions surrounding the sanctuary into automated sugarcane farms [24].
99 Therefore, the current study was conducted at DWS, one of the wildlife sanctuaries in southwest
100 Ethiopia, where scientific data are essential for studying the diversity and habitat association of
101 medium- and large-sized mammals. These studies serve as the foundation for additional
102 conservation and management strategies.

### 103 The study area and methods

104 **Description of the Study Area**
105 The study was conducted in Dhidhessa Wildlife Sanctuary (DWS) and its surrounding areas in
106 southwestern Ethiopia. The Sanctuary is located between 8^0^30’ and 8^0^40’ N latitude and between
107 36^0^22’ and 36^0^43’ E longitude and covers a total area of 1300 km^2^. The sanctuary is about 395
108 km southwest of Addis Ababa (the capital city) and shared among three administrative zones
109 (East Wollega, Buno Bedele, and Jimma), all within Oromia National Regional State (Fig. 1).
110 The altitude within the sanctuary ranges between 1350 and 1050 masl. The sanctuary has two
111 different seasons, dry (November to February) and wet (June to September), with uni-modal
112 rainfall ranging from 648 mm to 2001.8 mm. The sanctuary experienced relatively warm
113 temperatures, with mean annual minimum and maximum temperatures of 12°C and 35°C [25]. In
114 addition to the vast lowland plains along the Dhidhessa river bank, the land form of the sanctuary
115 includes undulating hilly terrain and mountains surrounding the sanctuary from the north and
116 western sides. Habitats in the study area include wooded grassland, riparian forests, seasonal
117 flooded prairies, and extensive savannah grassland. This region was once home to a variety of
118 wildlife species (especially antelopes, mega herbivores, and large carnivores). For the
119 conservation of the habitats and the large mammals therein, the Ethiopian government
120 designated the DWS in 1970. Before the management system of the sanctuary was well
121 established, for various reasons (including the political instability that spans over three decades
122 and the extensive settlement program for people displaced from the north and eastern parts of the
123 country), part of the then wildlife areas were taken and other parts were heavily encroached. In
124 addition, the extensive plain area adjacent to the sanctuary from the northeastern side was used
125 for the mechanized sugarcane farm to produce raw materials for the state-owned Arjo Dhidhessa
126 Sugar Factory (ADSF). As a result, some evaluative reports show that the DWS prematurely
127 hindered itself from achieving the objectives for which it was established [24].

### 128 Methods

129 A preliminary survey was carried out in February 2021 in the study area to secure information
130 such as the climatic conditions, habitat types, topographic information, level of anthropogenic
131 activities, etc. The Dhidhessa Wildlife Sanctuary (DWS) was characterized by heterogeneous
132 habitats. Based on the vegetation types, the habitat types of the study area were classified into
133 four (riparian forest, savanna grassland, seasonally flooded grassland and wooded grassland)
134 [2,26]. During the preliminary survey, sampled study sites were determined (Table 1).
135 Transect lines counts were used to determine the diversity, and habitat association of medium
136 and large sized mammals. Transect lines was placed on the stratified habitat types in a random
137 fashion and proportional to an area of the habitat type [27]. A total of 25 lines transects, covering
138 about 25% (24.65 km^2^) of the study area. Wildlife trails, footpaths, and other access routes were
139 also used as transect lines. The average length of line transect was five km and the width were
140 determined by the vegetation type and season, and ranges between 75 m (riverine and tall grass
141 bearing wooded grassland) and 200 m (open grasslands) and each transect ends about a km from
142 the habitat edge [28]. Adjacent transect lines were spaced between one and two Km.
143 Data collection was conducted once a month for a total of 6 months during both the dry seasons
144 (December, January and February, 2022) and the wet seasons (April, May and June, 2022) to
145 assess the diversity, and habitat association of medium and large sized mammals of DWS.
146 Mammal survey was carried out for three days per season and two times per day 8:00 a.m. to
147 10:00 a.m. in morning and 4:00 p.m. to 6:00 p.m. late afternoon, when most diurnal medium and
148 large mammals were more active in the study area [29-30]. Each transect line of each habitat
149 type was visited once for a month and at the same period (early morning and evening). During
150 data collection the researcher and 24 assistants person were walking on foot along transect line
151 established and directly count all the individuals of medium and large mammals sighted with
152 their respect species using naked eye and digital camera [31]. Upon detection of animals; habitat
153 types, season of the year, species name and number of individuals observed was record. For
154 identification of mammal species, local expertise and Kingdon Field Guide to African Mammals
155 [32] were used.
156 During the line transect survey, indirect evidences such as Bushnell wildlife camera traps, scats,
157 foot print & hoof and pug marks, hair remains, characteristic burrows, kill remains, etc., were
158 also used to determine the presence/absence of the species and their relative abundance [33]. All
159 basic information of the observed mammals (the species, group size, habitats types, the season,
160 etc.) were recorded.

### 161 Data analysis

162 All data collected during the study period were presented in a table by season and habitat type.
163 The Shannon–Wiener Diversity Index was used [34] to calculate the distribution, abundance, and
164 evenness of species between the wet and dry seasons and within habitat types, and SPSS version
165 20 software was used to analyze the data. Excel version 2016 was also used to generate the
166 relative abundance and species diversity indexes using formatted formulas. Moreover, the
167 conservation status of mammals identified from DWS was described as vulnerable and least
168 concerned as per the IUCN Red List [35]. A chi-square (χ2) test at 0.05 degree of
169 significance might be used to evaluate differences in the abundance of mammal species between
170 habitats and the standard seasonal distinction in the abundance of medium- and large-sized
171 mammalian species in the study area vicinity.

### 172 Results

173 **Species composition and richness**
174 In the study area, during the dry and wet seasons, a total of 27 species of medium- and large-
175 sized animals from five orders and twelve families were identified (Table 2). Of these, 1511
176 photographs taken with a camera trap over the research periods demonstrated the presence of 11
177 animal species (S1Fig.). Five orders—Primate, Artiodactyla, Carnivora, Rodentia, and
178 Tubulidentata—were used to group the recorded mammals. With eleven species, the order
179 Artiodactyla has the most of all the orders, followed by the order Carnivora with nine species.
180 Conversely, one species each represented the orders Rodentia and Tubulidentata. With seven
181 species, the Bovidae family was the largest family at the family level, followed by the
182 Cercopithecidae family. On the other hand, only one species represented each of the six families.
183 Of the 27 species of medium- and large-sized mammals found in the current study area, five
184 species—the crested porcupine (*Histrix cristata*), honey badger (*Mellivora capensis*), black-
185 blacked jackal (*Canis mesomelas*), common jackal (*Canis aureus*), and aardvark (*Orycteropus*
186 *afer*)—accounted for 18.5% of the total. The remaining 22 species, or 81.5 %, were large
187 mammals (Table 2).

## 188 Species relative abundance and conservation status of mammals

189 There were significant variations in the seasonal species abundance of medium- and large-sized
190 mammals, with a total of 2019 individuals reported, of which 1093 (54.1%) were observed
191 during the dry season and 926 (45.9%) individuals during the rainy season (χ2 = 13.81, df = 1,
192 p<0.05). *Papio anubis* represented (n = 500, 24.9%) was the most abundant species individuals
193 recorded followed by *Hippopotamus amphibious* (n = 364, 17.8%). On the other hand, *Civettictis*
194 *civetta* (n = 13, 0.64%) and *Panthera pardus* (n = 13, 0.64%) were the two least abundant
195 species (Table 3).
196 Out of the 27 species of mammals identified in DWS, 11 (40.7%) were considered occasional, 5
197 (18.5%) frequent, 4 (14.8%) common, and 7 (25.9%) rare. Comparably, Table 4 demonstrates
198 that of the known mammalian species, about 70.4% were categorized as least concern and 14.8%
199 as vulnerable. However, the remaining 14.8% are thought to be conservation-dependent, near
200 threatened, and at lower risk (Table 4).

### 201 Habitat association of mammals

202 In the research area, seasonal variations in the distribution of medium and large mammal species
203 were noted (Table 5). For example, mammals regularly used riparian forests and savanna
204 grasslands in both wet and dry seasons. In contrast, animals used the seasonal flooded grassland
205 (9.3%) less frequently in both seasons (Table 5). There was a noticeable difference in the
206 distribution of indicators linked to mammals’ observations in various habitat types during the
207 wet and dry seasons (*χ*2 = 18.87, df = 3, *p* < .05).

### 208 Species diversity and similarity indices

209 Table 6 shows that compared to other habitats, the Shannon diversity of mammal species was
210 higher in the savanna grassland (H = 2.44) and forest (H = 2.35). Conversely, seasonal flooded
211 grasslands had the lowest species diversity (H′ = 2.18).Nonetheless, there was no significant
212 variation in the Shannon-Wiener Index values among the four habitat types. Seasonally flooded
213 grasslands (E = 0.6849) and riparian forests (E = 0.4889) showed the highest and lowest
214 evenness of the mammalian species, respectively. On the other hand, in the riparian forest (D =
215 0.1482) and the savanna grassland (D = 0.1239), the highest and lowest dominance of
216 mammalian species were observed (S1 table). Additionally, during the dry and wet seasons, the
217 dominance of mammalian species and the Shannon different characteristics record were
218 comparable (S2 table). The research area's maximum species diversity (0.8761) was shown by
219 Simpson's index of diversity.

### 220 Discussion

221 **Diversity and relative abundance**
222 The topography, climate, and vegetation types of the current research area have produced a
223 variety of habitats for medium- and large-sized animal species. For example, the 27 species of
224 medium- and large-sized mammals found in the current study area had a significantly higher
225 diversity than the 23 species of mammals found in Arawale National Reserve, Kenya,[ 36], the
226 23 species found in the Baroye control hunting area [37], the 22 species found in the fragmented
227 remnant forest near Asella town [18], , a total of 18 species were observed by [2], around the
228 Wondo Genet forest area, 14 species of medium- and large-sized mammals were recorded in
229 Yayu forest by [38], and 12 species of mammals were identified in the Wabe forest's fragments
230 by [29]. A higher level of mammalian diversity in the current research area could have been
231 caused by sufficient vegetation cover and resource availability. Direct observations were also
232 used to identify the majority of the animal species found in the current research area. [23]
233 revealed that the Watch protected forest produced the same results. The fact that the habitats are
234 so open, maybe as a result of habitat loss and fragmentation, allows for the detection of
235 mammals with the unaided eye. The current study region has a high degree of secretiveness,
236 illusiveness, shyness, and human disturbance, which contributes to the low number of large
237 mammal species that were identified by indirect evidence rather than direct sighting. This
238 outcome was comparable to research conducted by [2] in Loka Abaya National Park and by [29]
239 from Wabe forest fragments.
240 In contrast to the high diversity of animals (41) documented in Gambella National Park, Ethiopia
241 [39] and 31 species recorded from Dhati Wolel National Park, Ethiopia [40] the results showed
242 comparatively lesser diversity in the study area. The lower diversity of mammals observed in the
243 current study area could be attributed to fragmentation caused by many anthropogenic activities,
244 severe habitat loss, conflict between humans and wildlife, or low level of attitude among local
245 residents toward conservations mammals [24]. Similarly, [41] noted that the demand for wide
246 areas, overexploitation, and habitat fragmentation have led to decline in medium- and large-sized
247 mammals.
248 The orders Artiodactyla (11 species) and Carnivora (9 species) were the most abundantly seen
249 mammal groups in the current study region. The findings of [40] in Dhati Wolel National Park
250 are consistent with the high number of Artiodactyla in the current research region. With the
251 largest diversity of ungulates on Earth, the African savanna environment has supported
252 multispecies animal production systems for thousands of years [42]. The large proportion of
253 riverine habitats and periodically flooded grasslands that supplied enough grass for grazing
254 during the dry season was probably responsible for the high diversity of herbivores in the current
255 study area. In addition, during the dry season, fires occur in the savanna grassland that is dotted
256 with Combertum-Terminalia woods. This would enable the high-nutrient new grasses to
257 regenerate, supporting a wide variety of grazers throughout the wet season. Rainfall, fire and
258 herbivory are the prime driving variables in African savanna ecosystems [42]. On the other
259 hand, a large number of different and prolific herbivore species are thought to be responsible for
260 the high diversity of carnivores seen in DWS. Large groups of herbivores, including
261 hippopotamus and African buffalo, were regularly seen throughout the current study, suggesting
262 their high abundance in the region and their ability to serve as a plentiful source of prey for a
263 variety of large carnivore species [43].

### 264 Relative abundance

265 In the current study area, during both the wet and dry seasons, *Papio anubis* was the species with
266 comparatively higher abundance. Nevertheless, the two least common species were *Civettictis*
267 *civetta* and *Panthera pardus*. Given that the species is suited to eating a wide range of foods and
268 endure various climatic and topographic differences, this may have something to do with its
269 feeding habit*s.* Similar finding was reported by [41] in Birbir protected forest western Ethiopia.
270 In contrast, carnivores were the least common in the study area in terms of both number and
271 distribution. According to respondents, the presence of illegal hunting as a result of widespread
272 livestock predation by carnivores (especially spotted hyena and common jackal) in and around
273 the research area significantly lowers their population. Because of human interference with their
274 cryptic and nocturnal behaviors, it was difficult to document their presence. Similarly, least
275 abundance of carnivores also reported in Dati Wolel National park [7].
276 Additionally, despite the fact that the area is surrounded by a landscape that is dominated by
277 humans and frequently poses a threat to their survival, many mammals have the ability to
278 transition from diurnal to nocturnal or crepuscular activity. In the riverine forest, however,
279 nonhuman monkey species were frequently sighted. This could be because nonhuman primates
280 have successfully adapted to human disturbance and changing environments. Similar finding was
281 reported by [44] in the Jorgo-Wato Protected Forest in Western Ethiopia.

### 282 Habitat association of mammals

283 Mammals in the current research region select one habitat over another depending on a variety of
284 factors, including soil types, topography, water availability, and the composition, quality, and
285 availability of feed [16, 23, and 45]. In the research area, riverine forest supported a large
286 number of mammal species during both seasons, followed by savanna grassland. Seasonally
287 flooded grasslands sustained the least amount of diversity. The abundance of food, water, and
288 vegetation cover may be the cause of this high observation rate, but the lack of refuges and
289 vegetation cover may be the reason for the low number of mammal individuals. Similar result
290 was reported by [16, 23, and 41]

### 291 Species diversity indices

292 The richness of species in the various ecosystems varies, as indicated by the species diversity
293 index. The savanna grassland in the current study region, for instance, has the highest species
294 diversity (H′=2.44), while the seasonal flooded grassland has the lowest species diversity
295 (H′=2.18). Likewise, studies by [18] showed that variables like habitat quality, species
296 preferences, availability of food resources, dense forest cover, and water are related to
297 variations in the distribution and abundance of mammal species within different habitats, and
298 that these variables are likely to lead to higher species richness. Our findings go counter to
299 those of the study by [23.46], which indicated that the natural forest had a greater species
300 variety. Human pressure on DWS is one potential source of these variations [24].

# 301 Conclusion and recommendations

302 The current study provided baseline data regarding the presence and abundance of medium- and
303 large-sized animal species in the DWS, southwestern Ethiopia, as well as information about
304 numerous anthropogenic activities. DWS is unique in that it is home to animals with great
305 conservation value, including large mammals like *Sincerus caffer* and *Hippopotamus*
306 *amphibibius*, and globally threatened species of mammals like *Panthera leo* and *Panthra pardus*,
307 which together indicate the area for biodiversity conservation. Twenty seven mammalian species
308 were identified in the study area. Order Artiodactyla had the highest number of species (11
309 species), followed by the orders Carnivora (9 species). While, orders Rodentia and Tubulidentata
310 were represented each by one species. Mammal species distribution and abundance in study area
311 vary due to vegetation types and resource availability. Riverine forest had the highest number of
312 species, followed by Savanna grassland. The distribution and utilization of different vegetation
313 communities by mammal species could be explained in terms of seasonal changes. The habitat
314 preferences of the medium and large-sized mammalian species were influenced by seasonal
315 variations in the quality and abundance of forage. It is possible to conclude that food, water, and
316 protection were decisive in determining the distribution of the mammal species in the present
317 study area. Currently, the pressure of intense human activity and improper farming practices
318 pose serious threats to the sustainability of wildlife of the area (S2 Fig.). Thus, study also
319 demonstrates the importance of DWS for broader biodiversity conservation and the requirement
320 for urgent actions that could rehabilitate preservation, management, and conservation of the
321 Sanctuary as well as local mammal species.

322

323

324

### 325 Supporting information

326 **S1: Appendix: Supplementary**
327 **Acknowledgements:** We thank to IDEA-WILD for their material support. We appreciate the
328 management of the Arjo Dhidhessa Sugar Factory. All the supports from the local communities
329 were also sincerely acknowledged.
330 **Authors’ contributions:** The study data collection was conceptualized and designed by Girma
331 Gizachew, Tadesse Habtamu, Tariku Mekonnen, Tibebu Alemu. Girma Gizachew did the
332 fieldwork, analyzed it, wrote the manuscript, and revised it all. Tadesse Habtamu, Tariku
333 Mekonnen and Tibebu Alemu edited the manuscript and amended the primary document's final
334 version before submitting it for evaluation. The authors participated in the manuscript's writing
335 and approved the final version.
336 **Data Availability:** All relevant data is within the paper and it’s supporting information files.
337 **Funding:** The author(s) received no specific funding for this work.
338 **Competing interests**: The authors declare no conflict of interest
339 **Ethics approval:** No approval of research ethics committees was required to accomplish the
340 goals of this study.

### 341 References

342 1. IUCN. (2019). IUCN Standards and Petitions Committee. Standards and Petitions Committee,

343	1(August), 1–60.

344 2. Zerihun Girma, Yosef Mamo, M. E. (2012). Species composition ,distribution and relative
345 abundance of large mammals in and around Woundo Genet forest patch,Southern
346 Ethiopia.
347 3. Diriba, G., Tamene, S., & Mengesha, Girma, A. A. (2020). *Diversity of medium and large*
348 *mammals in the Loka Abaya*. *June*, 9896–9905. https://doi.org/10.1002/ece3.6649
349 4. Tefera, M. (2011). Wildlife in Ethiopia: Endemic Large Mammals. World Journal of Zoology,

350	6(2), 108–116.

351 5. Amare, A. (2015). Wildlife Resources of Ethiopia: Opportunities, Challenges and Future
352 Directions: From Ecotourism Perspective: A Review Paper. *Natural Resources*, *06*(06),
353 405–422. https://doi.org/10.4236/nr.2015.66039
354 6. Lavrenchenko, L. A., & Bekele, A. (2017). Diversity and conservation of Ethiopian mammals:
355 What have we learned in 30 years? April 2018.
356 7. Rabira, G., Tsegaye, G., & Tadesse, H. (2015). The diversity, abundance and habitat
357 association of medium and large-sized mammals of Dati Wolel National Park, Western
358 Ethiopia. International Journal of Biodiversity and Conservation, 7(2), 112–118.
359 https://doi.org/10.5897/ijbc2014.0808
360 8. Bene, J. K., Bitty, E. A., Bohoussou, K. H., Abedi-, M., Gamys, J., & Soribah, P. A. J. (2013).
361 Current Conservation Status of Large Mammals in Sime Darby Oil Palm Concession in
362 Liberia. *Global Journal of Biology, Agriculture & Health Sciences*, *2*(3), 93–102.
363 9. Udy, K., Fritsch, M., Meyer, K. M., Grass, I., Hanß, S., Hartig, F., Kneib, T., Kreft, H.,
364 Kukunda, C. B., Pe’er, G., Reininghaus, H., Tietjen, B., Tscharntke, T., van Waveren, C.
365 S., & Wiegand, K. (2021). Environmental heterogeneity predicts global species richness
366 patterns better than area. Global Ecology and Biogeography, 30(4), 842–851.
367 https://doi.org/10.1111/geb.13261
368 10.Gebresenbet, F., Baraki, B., Yirga, G., Sillero-Zubiri, C., & Bauer, H. (2018). A culture of
369 tolerance: Coexisting with large carnivores in the Kafa Highlands, Ethiopia. Oryx, 52(4),
370 751–760. https://doi.org/10.1017/S0030605316001356
371 11. Reeder, D. M., Helgen, K. M., & Wilson, D. E. (2019). Global trends and biases in new
372 mammal species discoveries. Global Trends and Biases in New Mammal Species
373 Discoveries. https://doi.org/10.5962/bhl.title.156951
374 12.Wolf, C., & Ripple, W. J. (2018). Rewilding the world ’s large carnivores Subject Category :
375 Subject Areas : Author for correspondence : Royal Society Open Science, 5.
376 13. Ripple, W. J., Estes, J. A., Beschta, R. L., Wilmers, C. C., Ritchie, E. G., Hebblewhite, M.,
377 Berger, J., Elmhagen, B., Letnic, M., Nelson, M. P., Schmitz, O. J., Smith, D. W.,
378 Wallach, A. D., & Wirsing, A. J. (2014). Status and ecological effects of the world’s
379 largest carnivores. Science, 343(6167). https://doi.org/10.1126/science.1241484
380 14. Girma, Z., & Worku, Z. (2020). Large Mammal Diversity in Nensebo Forest, Southern
381 Ethiopia.	International	Journal	of	Zoology,	2020(December).
382 https://doi.org/10.1155/2020/8819019
383 15. Mengistu Wale, Abeje Kassie, Getachew Mulualem, Weldemariam Tesfahunegny, A. A.
384 (2017). Wildlife Threats and Their Relative Severity of Eastern Ethiopia Protected
385 Areas.	Ecology	and	Evolutionary	Biology,	2(4),	59.
386 https://doi.org/10.11648/j.eeb.20170204.12
387 16. Qufa, C. A., & Bekele, A. (2019). A preliminary survey of medium and large-sized mammals
388 from Lebu Natural Protected Forest, Southwest Showa, Ethiopia. Ecology and
389 Evolution, 9(21), 12322–12331. https://doi.org/10.1002/ece3.5733
390 17. Asefa, M., Cao, M., He, Y., Mekonnen, E., Song, X., & Yang, J. (2020). Ethiopian
391 vegetation	types,	climate	and	topography.	*Plant	Diversity*,	*42*(4),	302–311.
392 https://doi.org/10.1016/j.pld.2020.04.004
393 18. Kasso, M., & Bekele, A. (2017). Diversity , Abundance and Distribution of Mammals in
394 Fragmented Remnant Forests around Asella Town, Ethiopia. MAYFEB Journal of
395 Biology, 1(January), 1–12.
396 19. Belete Tilahun and Melese Merewa (2016). Assessment of large mammals potential in
397 Tululujia Wild life Reserve, South western Ethiopia
398 20. Ahumada, J., Faresin, C., Gajapersad, K., Hallam, C., Hurtado, J., Martin, E., McWilliam,
399 A., Mugerwa, B., O'Brien, T., Rovero, F., Sheil, D., Spironello, W., Winarni, N. and
400 Andelman, S. (2011). Community structure and diversity of tropical forest mammals:
401 data from a global camera trap network. Philosophical Transactions of the Royal Society
402 B: Biological Sciences. 366: 2703–2711.
403 21. Alemu, M. C. (2010). Mammalian Diversity in Borena-Sayint National Park, South Wollo,
404 Ethiopia. 1–74..
405 22. Dereje Negeri, Tsegaye Gadisa, Tadese Habtamu (2015). The Diversity, Distribution and
406 Relative Abundance of Medium and Large-sized Mammals in Baroye Controlled
407 Hunting Area, Illubabor Zone, Southwest Ethiopia. International Journal of Molecular
408 23. Geleta Mosissa & Bekele Afework. (2016). Survey of Medium and Large-sized Mammals in
409 Wacha Protected Forest , Western Ethiopia. Scholarly Journal of Agricultural Science,
410 6(3), 71–79.
411 24. Gizachew, G., Tefera, Tessema, T. H., & Bekere, Tibebu Alemu Bekere, T. M. G. (2024).
412 Human- common hippo ( Hippopotamus amphibius ) -conflict in the Dhidhessa Wildlife
413 Sanctuary	and	its	surrounding	,	Southwestern.	PLoS	ONE,	1–21.
414 https://doi.org/10.1371/journal.pone.0303647
415 25. NMA, 2022. National Meteorological Agency, Addis Ababa, Ethiopia.
416 26. Mamo, Y., Mengesha, G., Fetene, A., Shale, K., & Girma, M. (2016). Statu s of the Swayne ’
417 s Hartebeest , ( Alcelaphus buselaphus swaynei ) meta-population under land cover
418 changes	in	Ethiopian	Protected	Areas.	September	2012.
419 https://doi.org/10.5897/IJBC12.024
420 27. Varman, K. S., & Sukumar, R. (1995). The line transect method for estimating densities of
421 large mammals in a tropical deciduous forest: An evaluation of models and field
422 experiments.	Journal	of	Biosciences,	20(2),	273–287.
423 https://doi.org/10.1007/BF02703274 Evolution and Biodiversity, 5(4):1-9
424 28. Regassa, R., & Yirga, S. (2013). Distribution , abundance and population status of B urchell ’
425 s	zebra	(	Equus	quagga	)	in	Yabello	Wildlife.	5(3),	40–49.
426 https://doi.org/10.5897/JENE2012.0352
427 29. Legese, K., Bekele, A., & Kiros, S. (2019). A Survey of large and medium-sized mammals in
428 Wabe forest fragments, Gurage zone, Ethiopia. International International Journal of
429 Avian & Wildlife Biology, 4(2), 32–38. https://doi.org/10.15406/ijawb.2019.04.00149
430 30. Maíra Benchimol. (2016). Medium and Large-sized Mammals. Conservation International,
431 April, 207.
432 31. M.Norton-Griffiths. (1978). Norton-Griffith MN. (1978). Counting Animals (2nd edition).
433 African Wildlife Foundation, Nairobi, Kenya, pp.1-25. In Journal of Chemical
434 Information and Modeling (Vol. 53, Issue 9).
435 32. Kingdon, J. (2015). Kingdon field guide to African mammalian species-bloomsbury natural
436 history (2nd ed.).
437 33. Larsen, T. H. (2016). Core Standardized Methods for Rapid Biological Field Assessment.
438 Conservation International, June, 207.
439 34. Shannon, C. E., & Weaver, W. (1949). The mathematical theory of communication. Chicago,
440 IL: University of Illinois Press. (pp. 3–35).
441 35.IUCN.	(2019).The	IUCN	Red	List	of	Threatened	Species:

442	https://doi.org/10.2305/IUCN.UK.2019-2.RLTS.T41695A147992107.

443 36. Njoroge, P., Yego, R., Muchane, M., Githiru, M., Njeri, T., & Giani, A. (2009). A Survey of
444 the Large and Medium Sized Mammals of Arawale National Reserve, Kenya. Journal of
445 East African Natural History, 98(1), 119–128. https://doi.org/10.2982/028.098.0108
446 37. Negeri, D., Gadisa, T., & Habtamu, T. (2015). The Diversity, Distribution and Relative
447 Abundance of Medium and Large-sized Mammals in Baroye Controlled Hunting Area,
448 Illubabor Zone, Southwest Ethiopia. International Journal of Molecular Evolution and
449 Biodiversity. https://doi.org/10.5376/ijmeb.2015.05.0004
450 38. Gebrecherkos Woldegeorgis and Tilaye Wube. (2012). A Survey On Mammals Of The Yayu
451 Forest In Southwest Ethiopia. 35(2), 135–138.
452 39. UNEP-WCMC. (2010). UNEP-WCMC: Review of species/country combinations subject to
453 long-standing import suspensions: Mammal and bird species from Africa. UNEP-
454 WCMC, Cambridge. Pp. 109
455 40. Tesso, G. J. (2015). Large Mammal Diversity and the Ecology of African Buffalo (Syncerus
456 caffer Sparrman, 1779) in Dhati Wolel National Park, West Ethiopia. November.
457 41. Indiris Abdu, Gutema Jira and Gatriay Tut (2018).Diversity and Relative Abundance of
458 Medium and Large Mammals in Birbir Protected Forest, Western Ethiopia
459 42. Du toit, T. and Cumming, D.H. (1999). “Functional significance of ungulate diversity in
460 African	savannas	and	the	ecological	implications	of	the	spread	of
461 pastoralist.”Biodiversity Conservation, 8, 1643 ̶ 1661.

462

463 43. Legendre, P. and Legendre, L. (2012). Numerical Ecology, 3rd edn. Elsevier, Amsterdam.
464 44. Erena, M. G. (2021). Assessment of medium and large-sized mammals and their behavioral
465 response toward anthropogenic activities in Jorgo-Wato Protected Forest, Western
466 Ethiopia. *Ecology and Evolution*, *12*(2), 1–13. https://doi.org/10.1002/ece3.8529.
467 45. O’Kane C, Macdonald D. Seasonal influences on ungulate movement within a fenced South
468 African reserve. J Trop Ecol. 2018;34(3):200–3.
469 46.Gebo, B., Takele, S., & Shibru, S. (2021). Impacts of habitats and seasons on mammalian
470 diversity and distribution in the Faragosa-Fura landscape, Gamo Zone, Southern
471 Ethiopia.	*Geology,	Ecology,	and	Landscapes*,	*00*(00),	1–12.
472 https://doi.org/10.1080/24749508.2021.1944798

473

474

475

**Table 1.** Area of study and transect line

| 476 |  | | | | |
| --- | --- | --- | --- | --- | --- |
|  | Habitat type | Area (km^2^) |  | Number of sample  transects | Length and width of  the transects (km) |
|  | Riparian forest | 17.75 |  | 4 | 5x0.2 |
|  | Wooded grassland | 38.5 |  | 10 | 5x0.2 |
|  | Savanna grassland | 27.2 |  | 7 | 5x0.2 |
|  | Seasonally flooded grass land | 15.11 |  | 4 | 5x0.2 |
|  | Total | 98.56 |  | 25 |  |
| 477 |  |  |  |  |  |
| 478 | **Table 2:** Mammalian species | identified | in | study area (F = Foot | prints; S = Seen; P = |

479	Photographs; D = Droppings; B = Borrows, * = Community information)

Order	Family	Species	Common name	Local name (

Oromifa)

Sign

recorded

Primate	Cercopithecidae	*Cercopithecus*

*mittis Chlorcebus aethiops*

Blue monkey	Qamalee	F,S,P

Vervet monkey	Qamalee	S,P, F

*Papio anubis*	Anubis baboon	Jaldeesa	S,F,P

*Cercopethicus neglectus*

DeBrazzes monkey

Chano	S,P

Colobinae	*Colobus guerza*	Gureza	weenni	S,P,F

Artiodactyla	Suidae	*Phacochoerus africanus Potamochaerus*

*larvatus*

Warthog	Karkaroo	P,S,D

Bush pig	Booyye	S,P

*Hylochoerus meinertzhageni*

Giant forest hog

Booyye	P

Hippopotamidae	*Hippopotamus*

*amphibius*

Hippopotamus	Roobii	S,P,F,

Bovidae	*Syncerus caffer*	African buffalo	Gafarsa	S,P,F,D

*Tragelaphus scriptus*

Bush buck	Bosonu	P,S

*Sylvicapra grimmia*	Bush Duiker	Quruphe	P,F,D

*Kobus ellipsiprymnus Oreotragus oreotragus*

water buck	Worabbaa	*

Klipspringer	*

*Redunca redunca*	Reedbuck	*

*Ourebia ourebi*	Oribi	*

Carnivora	Felidae	*Panthera leo*	Lion	Leenca	F,D,*

*Panthera pardus*	Leopard	Qerrensa	P,D

*Leptailurus serval*	Serval cat	Iya	*

*Felis caracal*	Caracal	*

Hynaenidae	*Crocuta Crocuta*	Spotted Hyena	Warabessa	P,D

Mustelidae	*Mellivora capensis*	Honey badger	Amaaqexa	*

Viverridae	*Civettictis civetta*	African civet	Xirinyi	*

Canidae	*Canis aureus*	Common jackal

*Canis mesomelas*	Black blacked

jackal Rodentia	Hystricidae	*Histrix cristata*	Crested

porcupine

Wangoo	F,P

Jeedala	*

Dhaddee	P,S

Tubulidentata	Orycteropodidae	*Orycteropus afer*	Aardvark	Waldigessa	S

480

481	**Table 3:** Relative abundances of mammalian species recorded in the study areas

Species name	Number of individuals’ recorded	Relative abundance (%)

|  |  | Dry season | Wet season | Dry season | Wet  season | Total |
| --- | --- | --- | --- | --- | --- | --- |
|  | *Cercopithecus mittis* | 75 | 53 | 6.9 | 5.7 | 6.3 |
|  | *Chlorocebus aethiops* | 142 | 127 | 12.9 | 13.7 | 13.4 |
|  | *Papio anubis* | 261 | 239 | 23.9 | 25.8 | 24.9 |
|  | *Cercopethicus neglectus* | 28 | 17 | 2.56 | 1.84 | 2.2 |
|  | *Colobus guerza* | 87 | 79 | 7.96 | 8.5 | 8.23 |
|  | *Phacochoerus africanus* | 13 | 8 | 1.19 | 0.9 | 1.05 |
|  | *Hylochoerus* | 18 | 13 | 1.7 | 1.4 | 1.6 |
|  | *meinertzhageni* |  |  |  |  |  |
|  | *Potamochaerus larvatus* | 37 | 24 | 3.4 | 2.6 | 3 |
|  | *Hippopotamus amphibius* | 231 | 133 | 21.13 | 14.4 | 17.8 |
|  | *Syncerus caffer* | 28 | 45 | 2.56 | 4.9 | 3.7 |
|  | *Tragelaphus scriptus* | 32 | 24 | 2.93 | 2.6 | 2.8 |
|  | *Sylvicapra grimmia* | 23 | 12 | 2.1 | 1.3 | 1.7 |
|  | *Kobus ellipsiprymnus* | 12 | 6 | 1.09 | 0.65 | 0.9 |
|  | *Oreotragus oreotragus* | * | * | * | * | * |
|  | *Ourebia ourebi* | * | * | * | * | * |
|  | *Redunca redunca* | * | * | * | * | * |
|  | *Panthera leo* | 12 | 9 | 1.09 | 0.97 | 1.03 |
|  | *Panthera pardus* | 8 | 5 | 0.73 | 0.54 | 0.64 |
|  | *Leptailurus serval* | 16 | 27 | 1.5 | 2.9 | 2.2 |
|  | *Crocuta crocuta* | 11 | 36 | 1.01 | 3.9 | 2.4 |
|  | *Felis caracal* | * | * | * | * | * |
|  | *Mellivora capensis* | 9 | 14 | 0.8 | 1.5 | 1.2 |
|  | *Civettictis civetta* | 8 | 5 | 0.73 | 0.54 | 0.64 |
|  | Canis aureus | 12 | 10 | 1.09 | 1.08 | 1.08 |
|  | Canis mesomelas | * | * | * | * | * |
|  | Histrix cristata | 19 | 17 | 1.74 | 1.84 | 1.8 |
|  | Orycteropus afer | 11 | 23 | 1 | 2.48 | 1.74 |
|  | Total | 1093 | 926 | 100 | 100 | 100 |
| 482 |  |  |  |  |  |  |

483	**Table 4:** Occurrence of medium and large sized mammals in the study areas

Species name	Local name	Occurrence

category

IUCN

category

*Cercopithecus mittis*	Blue monkey	Frequent	VU *Chlorocebus aethiops*	Vervet monkey	Common	LC *Papio Anubis*	Anubis baboon	Common	LC *Cercopethicus neglectus*	DeBrazzes monkey	Frequent	LC *Colobus guerza*	Gureza	Common	LC *Phacochoerus africanus*	Warthog	Occasional	LR/LC

| *Hylochoerus meinertzhageni* | Bush pig | Frequent | LR/LC |
| --- | --- | --- | --- |
| *Potamochaerus larvatus* | Giant forest hog | Frequent | LR/LC |
| *Hippopotamus amphibius* | Hippopotamus | Common | VU |
| *Syncerus caffer* | African buffalo | Occasional | NT |
| *Tragelaphus scriptus* | Bush buck | Occasional | LC |
| *Sylvicapra grimmia* | Bush Duiker | Occasional | LR/LC |
| *Kobus ellipsiprymnus* | water buck | Occasional | LC |
| *Oreotragus oreotragus* | Klipspringer | Rare | LC |
| *Redunca redunca* | Reedbuck | Rare | LR/cd |
| *Ourebia ourebi* | Oribi | Rare | LR/cd |
| *Panthera leo* | Lion | Occasional | VU |
| *Panthera pardus* | Leopard | Rare | VU |
| *Leptailurus serval* | Serval cat | Rare | LC |
| *Crocuta crocuta* | Spotted Hyena | Frequent | LC |
| *Hyaena hyaena* | Striped hyena | Rare | NT |
| *Mellivora capensis* | Honey badger | Occasional | LC |
| *Civettictis civetta* | African civet | Occasional | LC |
| *Canis aureus* | Common jackal | Occasional | LC |
| *Canis mesomelas* | Black blacked jackal | Rare | LC |
| *Histrix cristata* | Crested porcupine | Occasional | LC |
| *Orycteropus afer* | Aardvark | Occasional | LC |

484	**Abbreviations:** LR/cd, Lower Risk/ conservation dependent; LR/LC, Lower Risk/ least concern;

485	LC, Least Concern; NT, Near Threatened; VU, Vulnerable

486

487	**Table 5:** Habitat occurrence (%) of large mammals during the wet and dry seasons in the study

488	area

Number of individuals observed in different habitats (%)

Season	Riparian forest	Wood land	Savanna grassland	Seasonal flooded

grassland

Dry	393 (36)	232 (21.2)	328 (30)	140(12.8)

Wet	339 (36.6)	252 (27.2)	287 (31)	48 (5.18)

Total	732 (36.3)	484 (23.97)	615 (30.5)	188 (9.3)

489

490

491

492

493	**Table 6:** Measuring of biodiversity (Index of diversity) in DWS

Habitats

|  | Variables | Riparian forest | Wooded  land | Savanna  grassland | Seasonal flooded  grassland |
| --- | --- | --- | --- | --- | --- |
|  | No. of species | 20 | 19 | 19 | 13 |
|  | No. individuals | 732 | 484 | 615 | 188 |
|  | Dominance_D | 0.1482 | 0.1339 | 0.1239 | 0.147 |
|  | Simpson_1-D | 0.8518 | 0.8661 | 0.8761 | 0.853 |
|  | Shannon_H | 2.28 | 2.346 | 2.44 | 2.186 |
|  | Evenness | 0.4889 | 0.5496 | 0.6036 | 0.6849 |
| 494 |  |  |  |  |  |
| 495 |  |  |  |  |  |
| 496 |  |  |  |  |  |

Manuscript	[Click here to access/download;Manuscript;Mammals manuscript](https://www2.cloud.editorialmanager.com/pone/download.aspx?id=37125487&guid=613f5e94-c7b3-48f9-89c6-2ff3230e34a4&scheme=1)[<https://www2.cloud.editorialmanager.com/pone/download.aspx?id=37125487&guid=613f5e94-c7b3-48f9-89c6-2ff3230e34a4&scheme=1> with anonymos.docx](https://www2.cloud.editorialmanager.com/pone/download.aspx?id=37125487&guid=613f5e94-c7b3-48f9-89c6-2ff3230e34a4&scheme=1)

Figure	[Click here to access/download;Figure;Fig 1.tif,Fig2.eps,etc..docx](https://www2.cloud.editorialmanager.com/pone/download.aspx?id=36949380&guid=db114cb3-86da-43c4-bb96-6c14b8d69387&scheme=1)[<https://www2.cloud.editorialmanager.com/pone/download.aspx?id=36949380&guid=db114cb3-86da-43c4-bb96-6c14b8d69387&scheme=1>](https://www2.cloud.editorialmanager.com/pone/download.aspx?id=36949380&guid=db114cb3-86da-43c4-bb96-6c14b8d69387&scheme=1)

**Figure 1: Map of study area** (Our team used shape files provided by the Ethiopian Mapping Agency to produce the map. The agency’s official website offers free access to these shape files [(http://www.ema.gov.et).](http://www.ema.gov.et/)

Supporting Information

[Click here to access/download](https://www2.cloud.editorialmanager.com/pone/download.aspx?id=36949382&guid=8c44fbaa-ec70-4ddf-aaa4-85795cc309f1&scheme=1)

[Supporting Information Supplementary.docx](https://www2.cloud.editorialmanager.com/pone/download.aspx?id=36949382&guid=8c44fbaa-ec70-4ddf-aaa4-85795cc309f1&scheme=1)
