# Supplementary material for: The diversity and habitat association of medium and large mammals in the Dhidhessa Wildlife Sanctuary, Southwestern Ethiopia
Source: PLoS One. 2025 Mar 21;20(3):e0317441. doi: 10.1371/journal.pone.0317441 (PMC11927904; doi:10.1371/journal.pone.0317441)
Supplement: Attachment — Submitted filename: renamed_008c6.docx [file pone.0317441.s004.docx]

**Date; - November, 7, 2024**

**To: Tunira Bhadauria, Ph.D.**

**Academic Editor**

**Journal of PLOS ONE**

**Subject: Responses to the reviewers’ comments**

**Dear Dr. Stephanie S. Romanach,**

We are very grateful for your and reviewers’ critical and constructive comments regarding our manuscript numbered **PONE-D-24-30486**, entitled *“The diversity and habitat association of medium and large mammals in the Dhidhessa Wildlife Sanctuary, Southwestern Ethiopia”.* Please find re-submitted (the revised version) of our manuscript in which we have carefully considered and addressed all comments raised by the referees. The revised version of the manuscript includes many improvements and summarized below. Moreover, the revised manuscript is presented using track-change with red color. We hope our responses are satisfactory and you will find this version suitable for publication in Journal of PLOS ONE. We look forward to hearing from you.

Dear Academic Editor, please note the following points

1. Our responses to the reviewers’ comments are provided
2. The line numbers with red colors (*line#)* refer to the improved version of the revised manuscript

Best regards,

Corresponding author

**Response to Academic editor #1**

1. **Comment 1(General comment),** the reviewers suggested that the manuscript when submitting your revision, we need you to address these additional requirements.
- The manuscript should meet PLOS One's style requirements.
- Data availability by the authors upon reasonable request.
- Figures 1 in your submission contain [map/satellite] images, which may be copyrighted.

***Response*:** Thank you very much for your suggestion. We agree with the comment and we made improvements to the issues mentioned above. However, we have already referenced Figure 1 on Line# 130.

2. **Comment 2,** **Line 1:** The reviewer one suggested that; - where is a short title in the title page? Explain abbreviations in the abstract section; do not repeat words or phrases from the topic directly at the keywords section. Please find equivalent words for them.

***Response*:** Thank you very much for your suggestion. We agree with the comment and made correction. Based on the comments and suggestions using a track change from Line# 32and 52 and we have re-written or revised the whole of portions the abstract. However, the guidelines of PLOS ONE stated that “short title” should be written in the submission system.

3. **Comment 3,** **Line 1:** The lack of data on habitat association and the absence of any ecological theory related to this concept.

***Response*:** Thank you very much for your comments and suggestions. We have made correction as commented on *Line#* 74-82.

4. **Comment 4,** **Line 59:** “Check and revise the number of mammalian species and their endemics in Ethiopia you mentioned in the introduction section“

***Response*:** Thank you very much for your suggestions. We have made correction as commented on Line# 60.

5. **Comment 5,** **Line 62:** Change the second paragraph into third paragraph and provide detail evidence on threats and level of impacts from global to local

***Response*:** Thank you very much for your comments and suggestions. We have changed and revised as commented *on* *Line#* 83-108.

6. **Comment 6, Line 102:** what is the objective of the study?

**Response:** Thank you very much for your suggestions. We agree with the comment and made correction by rewriting the words using a track change from Line# 121-123

7. **Comment 7, Line 104-120:** Detail information of the study area like map of the study area, historical background, loss of the area, purpose of establishment, and who is responsible to manage?

**Response:** Response: Thank you very much for your comments. We agree with the comment and made correction based on our objectives of the study from Line# 125-153

8. **Comment 8,** **Line 131:** List all the “etc” in the document

***Response*:** Thank you very much for your suggestions. We agree with the comment and made correction by rewriting the words using a track change from Line# 155-161

9. **Comment 9, Line 134 and 135:** Incomplete idea and make it on new paragraph

**Response:** Thank you very much for your suggestions. We have made correction on line #160 and 162

10. **Comment 10, Line 146-160:** Avoid repetition, clarity, how do they understand transect width to count spp. If not experts and uses separate sentence to explain how each indirect methods are used in your study.

**Responses:** Thank you very much for your comments. We agree with the comment and made correction., Based on the comments and suggestions using a track change from Line# 164-184, we have re-written or revised the whole of those portions.

11. **Comment 11**, **Line 164:** How you distributed the 25 transects in different habitat you used?

***Response*:** Thank you very much for your comments/suggestions. We have used based on area cover or size of the habitat (line #515).

12. **Comment 12, Line 165:** The whole area covers about 1300 km2 as you mentioned in the study area description above. How this calculation came? Does this percent of the sampled area represent the whole study area?

***Response*:** Thank you very much for your suggestions. The Ethiopian government formerly designed the Dhidhessa Wildlife Sanctuary in (line #138)1970 with the area 1300 km^2^ (line #128). However, at the moment, the area was used for various purposes. Based on these, we selected a sample from the whole 98.56 km^2^ (line #515) of the area; therefore, the 25% represents the sampled area rather than the whole area

13. **Comment 13,** **line 169:**   “How you avoided double counts of the animals?

***Response*:** Thank you very much for your comments. We agree with the comment and made correction *from Line#* 171-173 “As you indicated, in order to prevent double counting, adjacent transect lines were distributed one to two kilometers apart”.

14. **Comment 14,** **Line 170:** Does the month April the wet season in your study area?

Why not early morning at 6 am? Why you started from 8 am?

***Response*:** Thank you very much for your comments and suggestions. We agree with the comment and made correction using a track change *from* *Line#* 172-173.

15. **Comment 15, Line 515:** Show the total transects lines first before the actual transects you used under table 1.

***Response*:** Thank you very much for your comments. We agree with the comment and made correction using a track change *from* *Line#* 515.

16. **Comment 16,** **Line 162-171:** For analysis method use software

***Response*:** Thank you very much for your comments and suggestions. We agree with the comment and made correction using a track change *from* *Line#* 186-199 and 225 (as indicated that, we use PAST4 project software for the analysis of our study)

17. **Comment 17, Line 208:** “Not method provided”

***Response*:** Thank you very much for your suggestions. We agree with the comment and made correction *for* *Line#* 232 with track changes.

18. **Comment 18,** **Line 221:** The discussion section should follow logical flow of idea as it was presented in result section.

***Response*:** Thank you very much for your comments and suggestions. We agree with the comment and made correction using a track change *from* *Line#* 245, 292,310 and 320 (this section was re-written, as suggested).

19. **Comment 19,** **Line 238,240,249:**  How do you compare Sanctuary with National Park?
    ***Response*:** Thank you very much for your comments. However, we made the comparisons based on the methodologies and objectives rather than the area's size *from* *Line#* 249.
20. **Comment 20,** **Line 267,271:** Out of your study context, you didn’t use this method to collect data, as evidence lacks in method section.

***Response*:** Thank you very much for your comment. We agree with the comment and made correction using a track change *from* *Line#* 298-303 ("involving" re-write and re-worded as suggested).

21. **Comment 21,** **Line 296:** Include implication of the finding, not only summarizing, what is the specific recommendation based on your finding.

***Response:*** Thank you very much for your comment. We agree with the comment and made correction using a track change *from* *Line#* 330.

22. **Comment 22,** **Line 328:** Do you think this contribution only qualifies them for authorship? It’s very important to consider all valuable contributions.

***Response*:** Thank you very much for your comments and suggestions. We agree with the comment and made correction using a track change *from* *Line#* 365-369.

23. **Comment 23,** **Line 482:** change “occurrence”

***Response*:** Thank you very much for your suggestions. We have changed as commented, on #Line 531.

24. **Comment 24:** Use high resolution map of study area-300dpi.

***Response*:** Thank you very much for your comment. We agree with the comment and made correction.
